# A Novel Host-Proteome Signature for Distinguishing between Acute Bacterial and Viral Infections

**DOI:** 10.1371/journal.pone.0120012

**Published:** 2015-03-18

**Authors:** Kfir Oved, Asi Cohen, Olga Boico, Roy Navon, Tom Friedman, Liat Etshtein, Or Kriger, Ellen Bamberger, Yura Fonar, Renata Yacobov, Ron Wolchinsky, Galit Denkberg, Yaniv Dotan, Amit Hochberg, Yoram Reiter, Moti Grupper, Isaac Srugo, Paul Feigin, Malka Gorfine, Irina Chistyakov, Ron Dagan, Adi Klein, Israel Potasman, Eran Eden

**Affiliations:** 1 MeMed Diagnostics, Tirat Carmel, Israel; 2 Rambam Medical Center, Haifa, Israel; 3 Rappaport Faculty of Medicine, Technion-Israel Institute of Technology, Haifa, Israel; 4 Department of Pediatrics, Hillel Yaffe Medical Center, Hadera, Israel; 5 Department of Pediatrics, Bnai-Zion Medical Center, Haifa, Israel; 6 Faculty of Biology, Technion-Israel Institute of Technology, Haifa, Israel; 7 Applied Immune Technologies, Haifa, Israel; 8 Department of Internal Medicine, Bnai-Zion Medical Center, Haifa, Israel; 9 Infectious Diseases Unit, Bnai-Zion Medical Center, Haifa, Israel; 10 Faculty of Industrial Engineering and Management, Technion-Israel Institute of Technology, Haifa, Israel; 11 Pediatric Infectious Disease Unit and Clinical Microbiology Laboratory, Soroka Medical Center, Beer-Sheva, Israel; Kliniken der Stadt Köln gGmbH, GERMANY

## Abstract

Bacterial and viral infections are often clinically indistinguishable, leading to inappropriate patient management and antibiotic misuse. Bacterial-induced host proteins such as procalcitonin, C-reactive protein (CRP), and Interleukin-6, are routinely used to support diagnosis of infection. However, their performance is negatively affected by inter-patient variability, including time from symptom onset, clinical syndrome, and pathogens. Our aim was to identify novel viral-induced host proteins that can complement bacterial-induced proteins to increase diagnostic accuracy. Initially, we conducted a bioinformatic screen to identify putative circulating host immune response proteins. The resulting 600 candidates were then quantitatively screened for diagnostic potential using blood samples from 1002 prospectively recruited patients with suspected acute infectious disease and controls with no apparent infection. For each patient, three independent physicians assigned a diagnosis based on comprehensive clinical and laboratory investigation including PCR for 21 pathogens yielding 319 bacterial, 334 viral, 112 control and 98 indeterminate diagnoses; 139 patients were excluded based on predetermined criteria. The best performing host-protein was TNF-related apoptosis-inducing ligand (TRAIL) (area under the curve [AUC] of 0.89; 95% confidence interval [CI], 0.86 to 0.91), which was consistently up-regulated in viral infected patients. We further developed a multi-protein signature using logistic-regression on half of the patients and validated it on the remaining half. The signature with the highest precision included both viral- and bacterial-induced proteins: TRAIL, Interferon gamma-induced protein-10, and CRP (AUC of 0.94; 95% CI, 0.92 to 0.96). The signature was superior to any of the individual proteins (P<0.001), as well as routinely used clinical parameters and their combinations (P<0.001). It remained robust across different physiological systems, times from symptom onset, and pathogens (AUCs 0.87-1.0). The accurate differential diagnosis provided by this novel combination of viral- and bacterial-induced proteins has the potential to improve management of patients with acute infections and reduce antibiotic misuse.

## Introduction

Antibiotic overuse, typically stemming from use of these drugs to treat non-bacterial infections, has severe global health and economic consequences, including the emergence of antibiotic-resistant bacteria [1,2]. Antibiotic underuse due to delayed or missed diagnosis is also common (24%-40% of all bacterial infections) [3–6], and may result in prolonged disease state and medical complications [7–10]. A major cause of antibiotic overuse and underuse is the difficulty of distinguishing between bacterial and non-bacterial (mostly viral) etiologies [3,11]. While routinely used microbiological diagnostic tests such as culture, serology, and more recently nucleic acid based tests (e.g., RT-PCR) can assist the clinician in the etiological determination of the underlying infectious process, several challenges remain. These include: (i) pathogen detection in cases where the infection site is not readily accessible (e.g., pneumonia), or is unknown (e.g., fever without source); (ii) time to microbiological laboratory results may be lengthy (hours or days); (iii) interpreting whether a detected bacteria is the disease causing agent or a mere colonizer [12–14], and (iv) identification of a virus does not preclude the possibility that an undetected bacteria may be the cause of the underlying illness (i.e. a case of mixed infection with both virus and bacteria) [15,16].

An approach that has the potential to address these challenges relies on monitoring the host's immune-response to infection, rather than direct pathogen detection [17]. Recent studies examining host-RNA signatures in response to different infections show promising results [17–21]. However, quantitative and rapid measurements (within minutes) of multiple host-RNAs in the clinical setting remain a technical challenge, especially at the point-of-care where most antibiotics prescriptions are made. Circulating host-proteins are an attractive alternative as they can be rapidly measured at the point of need. Proteins that are routinely used to support diagnosis of infection include procalcitonin, C-reactive protein (CRP), and Interleukin-6 (IL-6) [22]. However, these markers are sensitive to inter-patient variability, including time from symptom onset, clinical syndrome, and pathogen species [22–27]. For example, multiple studies found that procalcitonin is valuable for guiding antimicrobial therapy duration and for predicting disease severity [28–30], however its diagnostic accuracy for detecting bacterial etiology in cases such as sepsis and pneumonia has been challenged [23,31–34]. Elevated CRP levels are suggestive of a bacterial infection [35], but similar levels may be observed in patients with some viral strains (e.g., adenovirus and influenza) [36], and inflammatory diseases. While IL-6 rises quickly in response to a bacterial trigger, its utility is dampened by a limited window of opportunity for measurement due to its rapid decay within approximately 36 hours [37].

To improve the performance of individual host-proteins, combinations of several proteins into a single predictive score have been proposed [11,38–43]. However, to date, in most studies these combinations provided only limited-to-moderate diagnostic improvement over individual proteins. This may be explained by the predominant focus on bacterial-induced proteins (e.g., procalcitonin, IL-6, IL-8, SAA, CRP and sTREM), which share biological pathways, and are thus inherently sensitive to the same factors. Therefore, we reasoned that the combination of unrelated host-proteins participating in different pathways may improve diagnostic accuracy. In particular, the inclusion of new host-proteins that are up-regulated in viral infections may be an innovative complement to bacterially-induced proteins in current clinical use.

Accordingly, the aims of the present study were threefold: to perform a large-scale screen of novel and traditional host-proteins; to integrate the findings into a computational signature for accurate diagnosis of acute bacterial (or mixed) versus viral infections; and to validate the signature on an independent cohort of patients.

## Materials and Methods

### Ethics Statement

We prospectively recruited 1002 patients between August 2009 and November 2013 from Hillel-Yaffe and Bnai-Zion Medical Centers, Israel (NCT01917461). The study was approved by the Hillel-Yaffe Medical Center Institutional Review Board (approval ID 0071–10-HYMC), and the Bnai-Zion Medical Center Institutional Review Board (approval ID 0084–12-BNZ). The study was conducted according to the guidelines and recommendations of Good Clinical Practice and the Declaration of Helsinki. Written informed consent was obtained from each participant or legal guardian, as applicable.

### Study population

**Fig 1:**
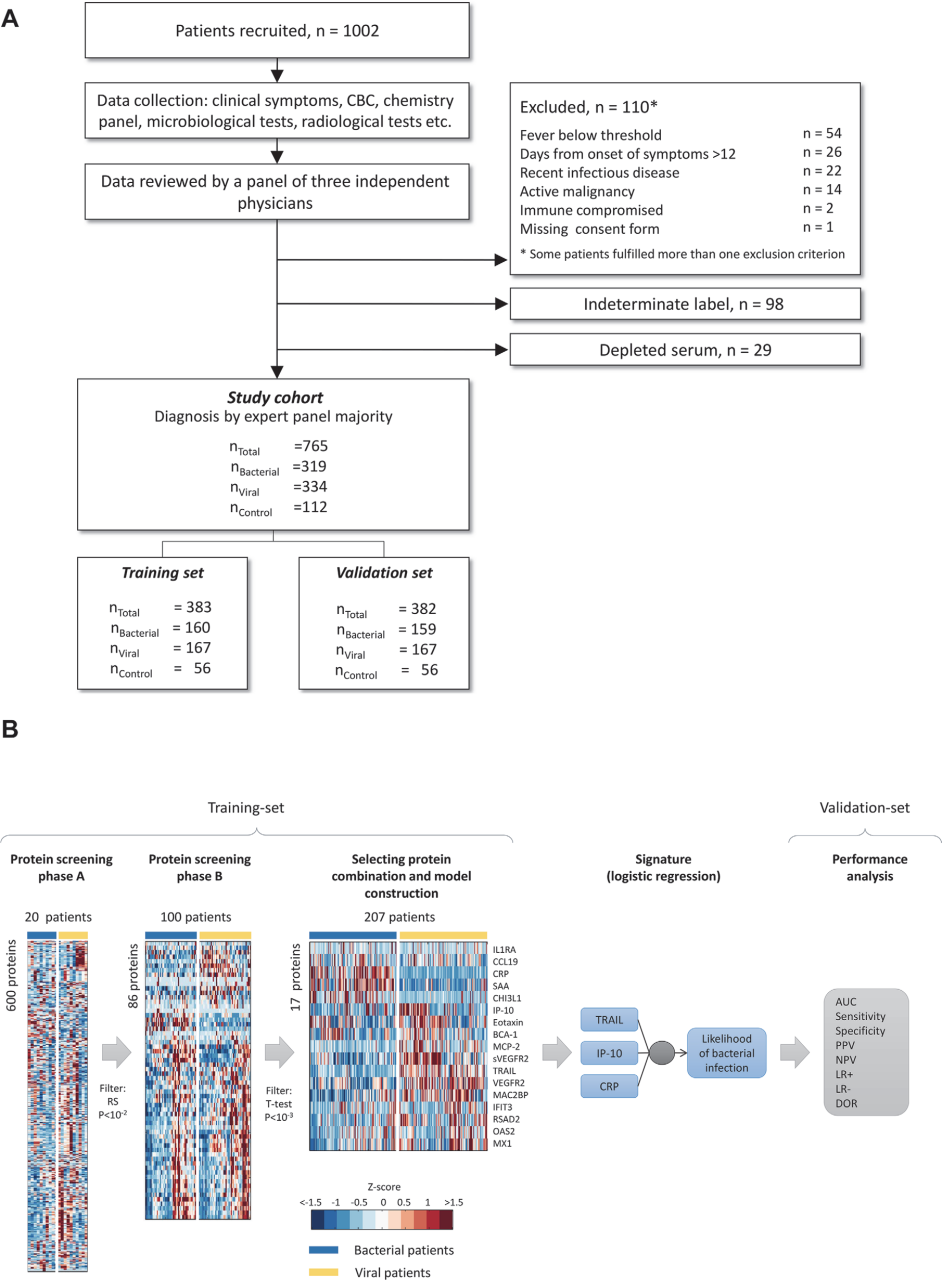
Study workflow. (A) An overview of the study workflow. n_Bacterial_, n_Viral_ and n_Control_ represent the number of bacterial (including mixed bacterial plus viral co-infections), viral and control (with no apparent infectious disease) cases, respectively. CBC—complete blood count. (B) An overview of protein screening, model construction and validation process.

Pediatric patients (≤18 years) were recruited from pediatric emergency departments, pediatric wards and surgical departments, and adults (>18 years) from emergency departments, internal medicine departments and surgical departments. Inclusion criteria for the infectious disease cohort included: clinical suspicion of an acute infectious disease, peak fever >37.5°C since onset of symptoms, and duration of symptoms ≤12 days. Inclusion criteria for the control group included: clinical impression of a non-infectious disease (e.g., trauma, stroke and myocardial infarction), or healthy subjects. Exclusion criteria included: evidence of acute infection in the two weeks preceding enrollment; congenital immune deficiency; treatment with immunosuppressive or immunomodulatory therapy; active malignancy; and human immunodeficiency virus (HIV)-1, or hepatitis B/C virus infection (Fig. 1A). Importantly, in order to enable broad generalizability, antibiotic treatment was not an exclusion criterion.

### Enrollment process and data collection

For each patient, the following baseline variables were recorded: demographics, medical history, physical examination, complete blood count obtained at enrollment, and chemistry panel. A nasal swab was obtained for microbiological investigation including two multiplex-PCR diagnostic assays (Seeplex RV15 [n = 713; 16 viral strains] and Seeplex PB6 [n = 633; six bacterial strains]), and a blood sample was obtained for host-protein measurements. Additional samples were obtained as deemed appropriate by the physician (e.g., urine [n = 420], stool [n = 66] and blood [n = 420] samples in cases of suspected urinary tract infection [UTI], gastroenteritis [GI] and bacteremia respectively) (S1 Data). Radiological tests were obtained at the discretion of the physician (e.g., chest X-ray for suspected lower respiratory tract infection [LRTI]). Thirty days after enrollment, disease course and response to treatment were recorded.

### Establishing the reference standard

For determining the infectious etiology we created a rigorous expert panel reference standard [44], which follows the broadly accepted recommendations of the Standards for Reporting of Diagnostic Accuracy (STARD)[45], and the NHS Health Technology Assessment (NHS-HTA) for evaluation of diagnostic tests [46]. First, we performed a thorough clinical and microbiological investigation for each patient as described above. This was followed by a review of the data collected throughout the disease course by a panel of three physicians. For adult patients the panel included the attending physician and two infectious disease specialists, while for children it included the attending pediatrician, an infectious disease expert and a senior attending pediatrician. Each panel member assigned one of the following diagnostic labels to each patient: (i) bacterial; (ii) viral; (iii) no apparent infectious disease or healthy (controls); and (iv) indeterminate. Patients diagnosed by the panel as having mixed infections (bacteria and virus) were labeled as bacterial because they are managed similarly. The panel members were blinded to the labeling of their peers to prevent group pressure or influential personality bias as recommended by NHS-HTA [46]. Importantly, they were blinded to the results of the signature. Final diagnosis was determined by panel majority (i.e. at least two of the three panel members assigned the same label) (Fig. 1A).

To examine the generalizability of the results it is recommended to examine whether alternative reference standard strategies affect performance [44,46]. Thus, two additional strategies were applied: (i) *Unanimous sub-cohort*: Patients were assigned the same label by all three panel members; and (ii) *Microbiologically confirmed sub-cohort*: Bacterial labeled patients were unanimously diagnosed by all three panel members and had one of the following microbiological confirmations: bacteremia (with positive blood culture), bacterial meningitis (with positive CSF culture), lower UTI (with positive urine culture), upper UTI (with positive urine culture and ultrasound demonstration of renal involvement) or peritonsillar abscess (proven by surgical exploration or computerized tomography). Viral labeled patients were unanimously diagnosed by panel members and had at least one laboratory detected virus.

### Samples, procedures and protein measurements

Venous blood samples were stored at 4°C for up to 5 hours, subsequently fractionated into plasma, serum and total leukocytes, and stored at -80°C. Nasal swabs and stool samples were stored at 4°C for up to 72 hours and subsequently transported to a certified service laboratory for multiplex PCRs. In the screening phase, host-proteins were measured using enzyme-linked immunosorbent-assay (ELISA), Luminex, protein-arrays and flow-cytometry. The three proteins used in the final signature were measured as follows: CRP using either Cobas-6000, Cobas-Integra-400/800, or Modular-Analytics-P800 (Roche); TRAIL and IP-10 using commercial ELISA kits (MeMed Diagnostics).

### Statistical analysis

Primary analysis was based on AUC, Sensitivity (TP/P), Specificity (TN/N), Positive likelihood ratio (LR+ = Sensitivity/[1-Specificity]), Negative likelihood ratio (LR− = [1–Sensitivity]/Specificity) and Diagnostic odds ratio (DOR = LR+/LR−), where P, N, TP and TN correspond to positives (bacterial patients), negatives (viral patients), true positives, and true negatives, respectively. Sample size calculations are presented in S2 Data. Half of the patients in the study were randomly assigned to the training-set (used for protein screening and model development), and the remaining half to a mutually exclusive validation-set (used to assess model performance). Importantly, none of the validation-set patients were used to train the algorithms, or to select the proteins (Fig. 1). A multinomial logistic regression model was used to integrate the levels of multiple proteins into a bacterial likelihood score. Patients whose probability of bacterial infection was intermediate, between 0.35 and 0.55, were flagged. We use the term 'equivocal immune response' to describe these patients because their profile borders between bacterial and viral host-responses. Statistical analysis was performed using MATLAB (MathWorks).

## Results

### Patient characteristics

Final diagnosis was attained in 765 patients (Fig. 1A). Additionally, 98 patients were labeled as indeterminate because disease etiology could not be established (Table 1). The cohort was balanced with respect to gender (47% females, 53% males) and included 56% pediatric patients (≤18 years) and 44% adults (>18 years). Patients presented with a wide range of clinical syndromes (e.g. RTI, UTI, and GI), maximal temperatures (36–41.5°C), time from symptom onset (0–12 days), comorbidities, and medications (Table 1 and S3 Data). Altogether, 56 pathogen species were detected that are responsible for the majority of acute infections in the Western world (S3 Data).

**Table 1:** Baseline characteristics of the study cohort patients.

**Criteria**		**Total**		**Children**		**Adults**	
				**(≤18 years)**		**(>18 years)**	
		**n = 765**		**n = 432**		**n = 333**	
**Age (years)**							
	<3	211	(28)				
	3–6	93	(12)				
	6–9	46	(6)				
	9–18	82	(11)				
	18–30	55	(7)				
	30–60	161	(21)				
	>60	117	(15)				
**Gender**							
	Female	363	(47)	205	(47)	158	(47)
**Maximal Temperature (°C)**							
	<37.5	106	(14)	28	(6)	78	(23)
	37.5–38.4	154	(20)	68	(16)	86	(26)
	38.5–39.4	294	(38)	164	(38)	130	(39)
	39.5–40.4	196	(26)	157	(36)	39	(12)
	>40.5	15	(2)	15	(3)	0	(0)
**Time from symptoms onset (days)**							
	0–1	175	(24)	118	(27)	57	(17)
	2–3	265	(36)	161	(37)	104	(31)
	4–5	161	(22)	89	(21)	72	(22)
	6–7	109	(15)	52	(12)	57	(17)
	8–9	10	(1)	2	(0.5)	8	(2)
	10–12	14	(2)	2	(0.5)	12	(4)
	N/A	31	(4)	8	(2)	23	(7)
**Clinical syndrome**							
	Cellulitis	28	(4)	7	(2)	21	(6)
	CNS	14	(2)	9	(2)	5	(2)
	GI	89	(11.5)	66	(15)	23	(7)
	LRTI	158	(21)	84	(19)	74	(22)
	Non-infectious	112	(14.5)	29	(7)	83	(25)
	Other	12	(1.5)	4	(1)	8	(2.5)
	Systemic	150	(19.5)	110	(26)	40	(12)
	URTI	145	(19)	104	(24)	41	(12)
	UTI	57	(7)	19	(4)	38	(11)
**Recruiting site**							
	Pediatrics & Internal	293	(38)	137	(32)	156	(47)
	PED & ED	472	(62)	295	(68)	177	(53)
**Hospitalization duration (days)**							
	Not hospitalized	272	(36)	174	(40)	98	(29)
	1–2	206	(28)	126	(29)	80	(24)
	3–4	170	(22)	94	(22)	76	(23)
	5–6	53	(7)	24	(6)	29	(9)
	7–8	31	(4)	7	(1.5)	24	(7)
	>8	33	(4)	7	(1.5)	26	(8)
**Season**							
	Autumn	181	(24)	111	(26)	70	(21)
	Spring	208	(27)	124	(29)	84	(25)
	Summer	170	(22)	98	(23)	72	(22)
	Winter	206	(27)	99	(23)	107	(32)
**Smoking**							
	Yes	74	(10)	0	(0)	74	(22)
	No	691	(90)	432	(100)	259	(78)
**Antibiotic prescription**							
	Yes	432	(56)	207	(48)	225	(68)
	No	333	(44)	225	(52)	108	(32)
**Detected microorganisms**							
	Not detected	219	(29)	79	(18)	140	(42)
***Viruses***							
	Adenovirus A/B/C/D/E	50	(7)	47	(11)	3	(1)
	Bocavirus 1/2/3/4	9	(1)	9	(2)	0	(0)
	CMV & EBV	25	(3)	23	(5)	2	(0.6)
	Coronavirus 229E/NL63/OC43	19	(2)	14	(3)	5	(2)
	Enteric viruses	19	(2)	16	(4)	3	(1)
	Enterovirus	21	(3)	20	(5)	1	(0.3)
	Influenza A virus	45	(6)	24	(6)	21	(6)
	Influenza B virus	19	(2)	14	(3)	5	(2)
	Metapneumovirus	17	(2)	13	(3)	4	(1)
	Parainfluenza 1/2/3/4	48	(6)	41	(9)	7	(2)
	Respiratory syncytial virus A/B	40	(5)	38	(9)	2	(0.6)
	Rhinovirus A/B/C	87	(11)	73	(17)	14	(4)
***Bacteria***							
	Atypical bacteria	27	(4)	7	(2)	20	(6)
	E.coli	44	(6)	17	(4)	27	(8)
	Enterococcus faecalis	10	(1)	0	(0)	10	(3)
	Group A Strep	19	(2)	16	(4)	3	(1)
	Haemophilus influenzae	179	(23)	148	(34)	31	(9)
	Streptococcus pneumoniae	306	(40)	207	(48)	99	(30)

Values are presented as total numbers, followed by the corresponding percentages in brackets. Only microorganisms that were detected in more than five patients are presented. CNS- central nervous system, GI—gastroenteritis, LRTI—lower respiratory tract infection, UTRI—upper respiratory tract infection, UTI—urinary tract infection, N/A—healthy controls or patients in which data was not obtained. Influenza A subgroup included H1N1 strains. The atypical bacteria subgroup included *Chlamydophila pneumoniae*, *Mycoplasma pneumonia* and *Legionella pneumophila*. The Enteric viruses subgroup included Rota virus, Astrovirus, Enteric Adenovirus and Norovirus G I/II. In the clinical syndrome analysis the LRTI group included pneumonia, bronchiolitis, acute bronchitis, and laryngitis; the URTI group included pharyngitis, acute otitis media, acute sinusitis and acute tonsillitis.

### Host-protein screening and signature development

**Fig 2:**
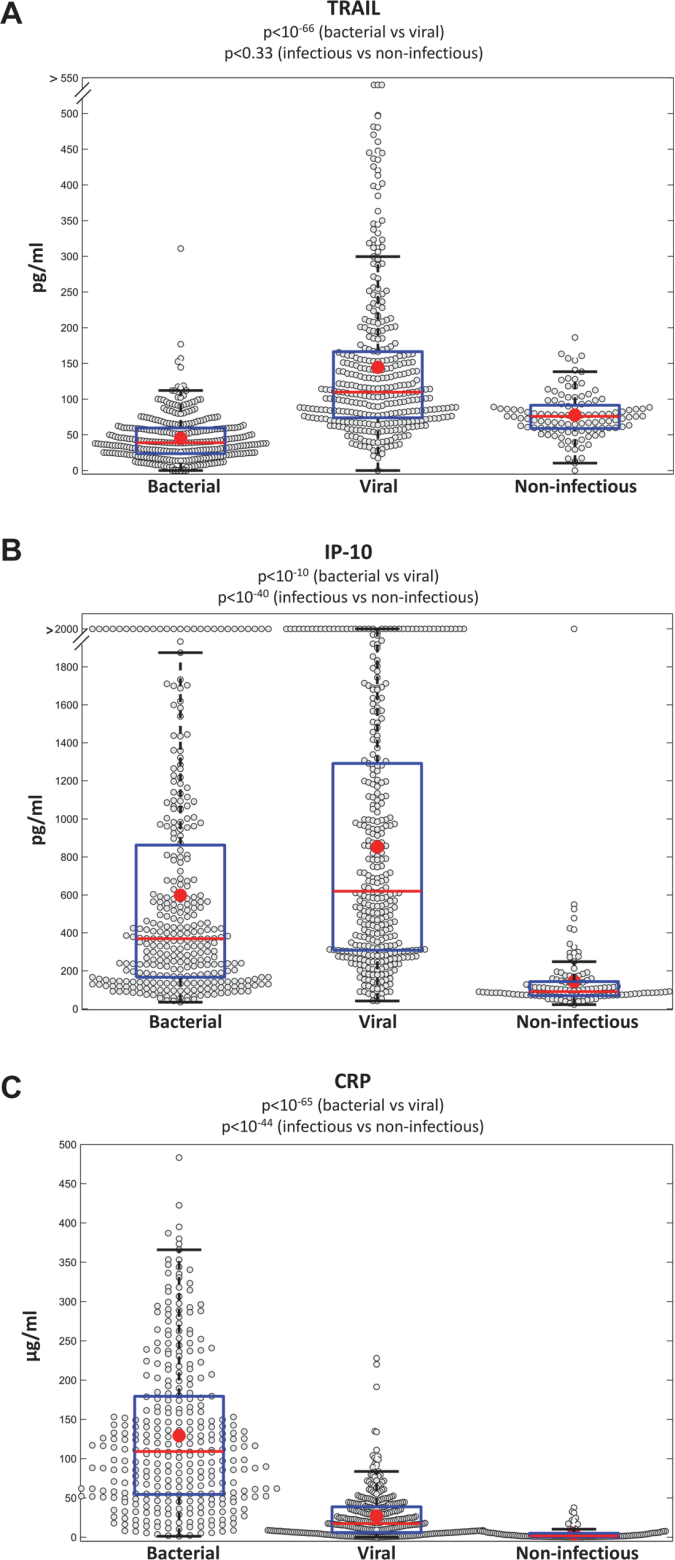
The proteins TRAIL, IP-10 and CRP are differentially expressed in bacterial, viral and non-infectious patients. Box plots for TRAIL (A), IP-10 (B), and CRP (C), measured over the entire study cohort (n = 765) are presented. Red line and circle correspond to group median and average respectively; t-test p-values between bacterial and viral groups and between infectious (bacterial and viral) vs non-infectious (including healthy subjects) are depicted.

A general overview of the process for developing, training and validating the multivariate logistic model is depicted in Fig. 1B. We performed a systematic literature and bioinformatics screening to identify 600 proteins that may be differentially expressed in response to infection (S2 Data). Next, each protein candidate was measured on 20–30 patients from the training-set (50% viral, 50% bacterial) and a Wilcoxon rank-sum *P*-value<0.01 was used to screen 86 proteins exhibiting statistically significant differential expression. Each of these was then evaluated in 100 additional patients (50% viral, 50% bacterial) and further screened using a t-test *P*<10^–3^ and a Bonferroni correction for multiple hypothesis testing, enabling downselection to 17 proteins (Fig. 1B). Multiple feature selection algorithms and computational models were evaluated to identify the optimal combination out of the final 17 proteins (S2 Data). The most discriminative signature (AUC of 0.95±0.03 attained on the training-set) used logistic regression to integrate the levels of the following viral- and bacterial-induced proteins (Fig. 2): TRAIL (member of the tumor necrosis factor family implicated in programmed cell death) [47], IP-10 (small cytokine implicated in multiple cellular processes including chemotaxis and cell growth inhibition) [48], and CRP (acute phase protein with diverse roles in tissue injury, infection and other inflammation processes) [49].

### Signature performance on the validation-set

We evaluated the signature performance using an independent validation-set that was neither used to screen the host-proteins nor to develop the signature. The AUC for distinguishing between bacterial and viral infections for the validation-set was 0.94±0.04. Similar results were obtained using leave-10%-out cross-validation on the entire cohort (AUC = 0.94±0.02), which supports signature generalizability (Table 2). The signature significantly outperformed all the individual proteins evaluated in the screening phase (*P*<10^–6^).

**Table 2:** Signature measures of accuracy for diagnosing bacterial vs viral infections.

	**All patients**			**Equivocal immune response filter**		
**Accuracy measure**	**Study cohort**	**Unanimous sub-cohort**	**Microbiologically confirmed sub-cohort**	**Study cohort**	**Unanimous sub-cohort**	**Microbiologically confirmed sub-cohort**
**AUC**	0.94 (0.92, 0.96)	0.96 (0.94, 0.98)	0.96 (0.92, 1.00)	0.94 (0.92, 0.96)	0.97 (0.95, 0.99)	0.97 (0.93, 1.00)
**Total accuracy**	0.88 (0.85, 0.90)	0.90 (0.87, 0.92)	0.91 (0.87, 0.94)	0.91 (0.88, 0.94)	0.93 (0.9, 0.96)	0.92 (0.88, 0.96)
**Sensitivity**	0.87 (0.83, 0.91)	0.88 (0.84, 0.91)	0.91 (0.84, 0.98)	0.92 (0.88, 0.96)	0.94 (0.9, 0.98)	0.95 (0.88, 1.00)
**Specificity**	0.90 (0.86, 0.93)	0.92 (0.89, 0.96)	0.90 (0.85, 0.95)	0.89 (0.86, 0.93)	0.93 (0.9, 0.96)	0.91 (0.87, 0.96)
**LR+**	8.7 (6, 12)	11.0 (7, 16)	9.1 (6, 14)	8.4 (6, 12)	13.4 (8, 21)	10.6 (6, 17)
**LR−**	0.14 (0.11, 0.19)	0.13 (0.09, 0.18)	0.1 (0.05, 0.21)	0.09 (0.06, 0.13)	0.07 (0.04, 0.11)	0.05 (0.02, 0.16)
**DOR**	60 (37, 98)	84 (47, 150)	91 (35, 239)	93 (53, 164)	208 (99, 436)	192 (55, 669)

Left: Performance estimates and their 95% CIs were obtained using a leave-10%-out cross-validation on all patients in the study cohort (n_Bacterial_ = 319, n_Viral_ = 334), Unanimous sub-cohort (n_Bacterial_ = 256, n_Viral_ = 271), and Microbiologically confirmed sub-cohort (n_Bacterial_ = 68, n_Viral_ = 173). Right: The analysis was repeated after filtering out patients with an equivocal immune response (study cohort [n_Bacterial_ = 290, n_Viral_ = 277, n_equivocal_ = 86], Unanimous [n_Bacterial_ = 233, n_Viral_ = 232, n_equivocal_ = 62] and Microbiologically confirmed [n_Bacterial_ = 64, n_Viral_ = 160, n_equivocal_ = 17]), which resembles the way clinicians are likely to use the signature. Additional measures of accuracy, including positive predictive value and negative predictive value, and their dependency on bacterial prevalence are described in S5 Data.

Next, we used the signature to distinguish between infectious (bacterial or viral) and non-infectious controls, which yielded an AUC of 0.96±0.02 for the validation-set. Further evaluation using leave-10%-out cross-validation confirmed the results (AUC = 0.96±0.01), outperforming any of the individual proteins (*P*<10^–8^).

### Signature performance is robust across different patient subgroups

**Fig 3:**
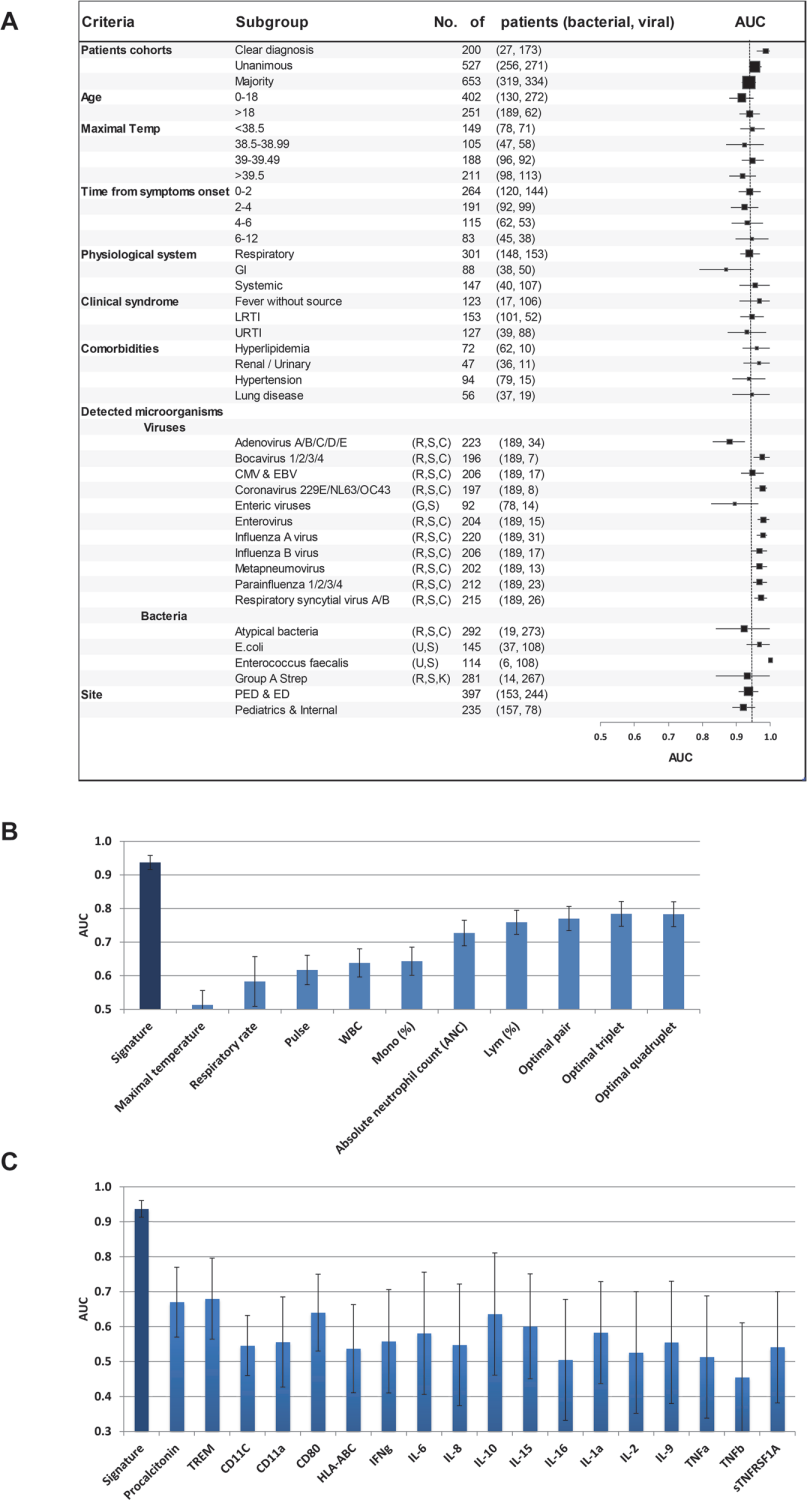
Signature performance is robust across different patient subgroups and outperforms lab parameters and protein biomarkers. (A) Signature AUCs in subgroups of the study cohort (bacterial and viral) are depicted. Square size is proportional to number of patients and error bars represent 95% CI. In the Pathogens analysis, each virus was compared to bacteria affecting the same physiological system, indicated in brackets. R-respiratory, C-central nervous system, G-gastrointestinal, U-urinary, K-skin, S-systemic (i.e. non-localized). Only pathogens detected in more than 5 patients are presented. PED—pediatric emergency departments, ED—emergency departments. For subgroup definitions see Table 1 legend. (B) Performance of clinical and lab parameters as well as the best performing pair (ANC and Lym %), triplet (ANC, Lym % and Pulse), and quadruplets (ANC, Lym %, Pulse, Mono %) of parameters, the values of which were combined using a logistic regression. Comparison was done on the entire study cohort (n = 653), apart from pulse (recorded in 292 bacterial and 326 viral patients), and respiratory rate (recorded in 292 bacterial and 326 viral patients). The signature performed significantly better (*P*<10^–15^) than the optimal quadruplet. (C) The signature performed significantly better (*P*<10^–8^) than biomarkers with a well-established role in the host response to infections. For each of the select biomarkers, analysis was performed in a subgroup of the study cohort (43≤n≤154 for each analysis, a convenience sample, n depended on the strength of the signal). Error bars represent 95% CI.

To examine whether the signature can maintain steady performance despite patient-to-patient variability, we performed subgroup analyses. The signature robustly distinguished between bacterial and viral infections (AUCs between 0.87 and 1.0) across a wide range of patient characteristics, including age, clinical syndrome, time from symptom onset, maximal temperature, comorbidities and pathogen species (Fig. 3A and S4 Data).

Next, we analyzed the degree to which alternative strategies for determining the reference standard affect performance [44,46]. Two strategies were applied. First, the Unanimous sub-cohort, whereby patient diagnosis was unanimously assigned by all three panel experts (n_Total_ = 639, n_Bacterial_ = 256, n_Viral_ = 271, n_Control_ = 112), yielded an AUC of 0.96±0.02 using leave-10%-out cross-validation. Second, the Microbiologically confirmed sub-cohort (n_Total_ = 353, n_Bacterial_ = 68, n_Viral_ = 173, n_Control_ = 112) yielded an AUC of 0.96±0.04.

To ensure conservative performance estimates and generalizability of results the analysis that follows focuses on the entire study cohort, rather than on the Unanimous or Microbiologically confirmed sub-cohort, which showed slightly improved performances.

### The Signature outperforms laboratory measurements, clinical parameters, and well-established biomarkers

We compared the performance of the signature to that of well-established clinical parameters and laboratory measurements, including white blood count (WBC), absolute neutrophil count (ANC), percentage neutrophils, maximal temperature, pulse, and respiratory rate (Fig. 3B and S6 Data). The signature surpassed all individual parameters (*P*<10^–18^). Next, we compared the signature to combinations of clinical parameters, which were developed using the same computational techniques used to construct the signature. The best performing pair, triplet and quadruplet are depicted in Fig. 3B (adding a fifth parameter did not improve performance). The signature surpassed the best performing combination of clinical parameters (AUC = 0.94±0.02 vs 0.77±0.04, *P*<10^–15^), which comprised ANC, pulse, % lymphocytes and % monocytes. Next, we compared the performance of the signature to that of procalcitonin and CRP, two proteins used in clinical practice to diagnose bacterial infections. The signature performed significantly better than both proteins and their combination (P<10^–8^, P<10^–6^, P<10^–6^ respectively; S6 Data). The signature also performed better than a wide range of host-proteins with an established role in the immune response to infection, including bacterial-related (e.g. TREM, IL-6 and IL-8), virus-related (e.g. IFN-γ and IL-2), and inflammation-related (e.g. IL-1a and TNF-α) proteins (*P*<10^–8^) (Fig. 3C and S6 Data).

### Signature performance is unaffected by the presence of potential colonizers

Many disease-causing bacteria are also part of the natural flora, and are frequently found in asymptomatic subjects [12–14]. Such bacteria pose a considerable diagnostic challenge, because merely detecting them does not necessarily imply a causative role in the disease; therefore, appropriate treatment may remain unclear. Accordingly, we asked whether the performance of the signature is affected by the presence of colonizers.

*Streptococcus pneumoniae* (SP) and *Haemophilus influenzae* (HI), detected by PCR on nasal swabs, were the two most common bacteria found in the study group (Table 1). High rates of SP and HI were observed amongst both bacterial and viral patients (SP: 36% and 47%; HI: 20% and 32%), substantiating the understanding that their mere presence does not necessarily cause disease [12]. We stratified the patients based on whether or not they had SP (SP+: n_Bacterial_ = 116, n_Viral_ = 157; SP-: n_Bacterial_ = 203, n_Viral_ = 177) and compared the AUC performance of the two groups. We did not observe a significant difference (0.93±0.03 vs 0.94±0.02, P = 0.31). The presence or absence of HI did not affect signature performance either (0.94±0.04 vs 0.93±0.02; HI+: n_Bacterial_ = 63, n_Viral_ = 106; HI-: n_Bacterial_ = 256, n_Viral_ = 228, P = 0.34). This indicates that the signature is unaffected by potential carriage of SP and HI.

## Discussion

The host-protein signature presented here addresses several challenges of current microbiological testing. (i) The difficulty of diagnosing patients whose infection site is inaccessible or unknown. The signature accurately diagnosed such cases, including LRTI (AUC 0.95±0.03, n = 153) and fever without source (AUC = 0.97±0.03, n = 123). (ii) Prolonged time to results (hours to days). The signature measures soluble proteins, which can be quantitated using an ELISA format. Moreover, these proteins are readily amenable to rapid measurement (within minutes) using hospital-deployed automated immunoassay machines and point-of-care devices. (iii) Identification of a virus does not exclude the possibility of mixed infection involving an undetected bacteria [15,16]. The signature classifies mixed infections with pure bacterial infections, thus prompting physicians to manage both groups similarly with regard to antibiotic treatment. (iv) A significant drawback of microbiological tests, PCRs in particular, is the detection of potential colonizers in subjects with non-bacterial diseases [12–14]. The signature performance was unaffected by the presence or absence of potential colonizers.

**Fig 4:**
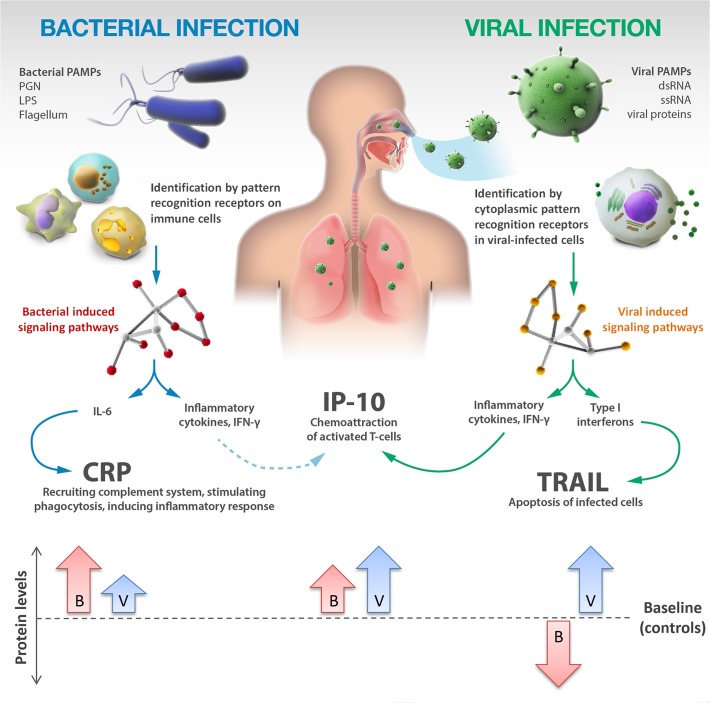
TRAIL, IP-10 and CRP participate in different signaling pathways and exhibit complementary dynamics in response to bacterial (B) and viral (V) infections. PAMPs—pathogen-associated molecular patterns; PGN—peptidoglycan; LPS—lipopolysaccharide.

The proteins included in the signature are not limited to well-established bacterial biomarkers. Six hundred host-proteins were screened (Fig. 1B), which to the best of our knowledge, is the largest quantitative proteome screening of patients with acute infection to date. Bioinformatics analysis was then used to identify a combination of three proteins with complementary dynamics: (i) We found that TRAIL was induced in response to a wide range of viral infections and surprisingly, also exhibited significantly reduced levels in bacterial infected patients (Fig. 2 and 4). Such opposing dynamics make TRAIL particularly suitable for distinguishing between bacterial and viral infections. The increase in viral patients may be related to TRAIL's role in programed cell death, which is a mechanism of infected cells to inhibit the virus from spreading [47]. The underlying reason for its reduction in bacterial infections warrants further studies. (ii) IP-10 levels were highly elevated in viral patients and less elevated in bacterial patients (Fig. 2 and 4). (iii) CRP levels were highly elevated in bacterial patients and less elevated in viral patients (Fig. 2 and 4). While the utility of increased CRP levels to suggest bacterial infections is well established [49], viral-induced proteins such as TRAIL are not used in clinical practice. The combination of novel viral-induced proteins that complement routinely used bacterial-induced proteins, substantially contributed to the signature’s robustness across a wide range of subgroups.

Currently, there is no single perfect reference standard for diagnosing bacterial and viral infections in a large fraction of the patients with symptoms of an acute infection. Several strategies can be applied, each with strengths and limitations [46]. Clinical suspicion confirmed by positive microbiological lab results is often used as a reference standard. However, limitations include false-positives due to technical issues (e.g., culture contamination [50,51] and uncertainty due to viral and bacterial carriage [12–14]). Moreover, these patients may represent the more severely ill, and potentially easier to diagnose cases, leading to overly optimistic performance. The expert panel diagnosis is another widely used approach [44,46]. It has the advantage of capturing a wider spectrum of illness severities including difficult-to-diagnose cases, and is therefore likely to generalize well to clinical practice [44,46]. However, the absence of a microbiological confirmation may lead to higher rates of mislabeling. Since these methods complement each other we applied both. A microbiologically confirmed reference standard was used for proof-of-principle on a sub-cohort of the patients yielding particularly high performance (AUC = 0.96±0.02), and the expert panel reference standard was used to generalize the results to the entire patient cohort (AUC = 0.94±0.02). The latter results are more conservative and likely to represent the performance of the signature in the real clinical setting.

A potential limitation of the current study is the heterogeneity of the patient cohort (multiple clinical syndromes, pathogen species, and time from onset of symptoms). We deliberately selected a diverse cohort in order to identify a signature that is insensitive to the inter-patient variability typically encountered in clinical settings. However, this diversity makes it more challenging to control for confounding factors. Although we did not identify significant confounders, follow-up studies on homogenous subgroups are warranted.

Finally, the present signature has broader applications beyond the scope of the present study that warrant further investigation. Future studies should seek to replicate our findings with patients of different ethnic populations, particularly in countries with a high prevalence of endemic chronic pathogens (e.g. *Mycobacterium tuberculosis*, *Plasmodium* and HIV). Follow-up time course studies that assess whether the signature can predict response to treatment and patient prognosis are also warranted.

Despite advances in infectious disease diagnosis, timely identification of bacterial infections remains challenging, leading to antibiotic misuse with its profound health and economic consequences. To address the need for better treatment guidance, we developed and validated a signature that combines novel and traditional host-proteins for differentiating between bacterial and viral infections. Our finding in a large sample size of patients is promising. The translational benefit could have a broad impact on the diagnosis and management of patients with acute infections.

## Supporting Information

S1 Data Microbiological investigation. (PDF)

S2 Data Data analysis and statistical methods. (PDF)

S3 Data Cohort characteristics. (PDF)

S4 Data The signature performance remains robust across different patient subgroups. (PDF)

S5 Data Performance analysis as a function of the prevalence of bacterial infections. (PDF)

S6 Data Comparison of the signature with alternative diagnostic methods. (PDF)
