# Supplementary material for: A Novel Host-Proteome Signature for Distinguishing between Acute Bacterial and Viral Infections
Source: PLoS One. 2015 Mar 18;10(3):e0120012. doi: 10.1371/journal.pone.0120012 (PMC4364938; doi:10.1371/journal.pone.0120012)
Supplement: S1 Data — (PDF) [file pone.0120012.s001.pdf]

1. Supporting Information S1 - Microbiological investigation

Patients underwent two multiplex-PCR diagnostic assays from nasal swab samples: (i) Seeplex® RV15 (n=713), for detection of parainfluenza virus 1, 2, 3, and 4, coronavirus 229E/NL63, adenovirus A/B/C/D/E, bocavirus 1/2/3/4, influenza virus A and B, metapneumovirus, coronavirus OC43, rhinovirus A/B/C, respiratory syncytial virus A and B, and Enterovirus, and (ii) Seeplex® PB6 (n=633) for detection of *Streptococcus pneumoniae*, *Haemophilus influenzae*, *Chlamydophila pneumoniae*, *Legionella pneumophila*, *Bordetella pertussis*, and *Mycoplasma pneumoniae*. Multiplex-PCR assays were performed by a certified service laboratory. Patients were also tested for additional pathogens according to their suspected clinical syndrome, including: blood culture (n=420), urine culture (n=188) and stool culture for *Shigella spp*., *Campylobacter spp*. and *Salmonella spp*. (n=66); serological testing (IgM and/or IgG) for cytomegalovirus (CMV), Epstein-Barr virus (EBV), *Mycoplasma Pneumonia*, and *Coxiella burnetii* (Q-Fever) (n=167, n=130, n=206 and n=41 respectively).
