# Supplementary material for: A Novel Host-Proteome Signature for Distinguishing between Acute Bacterial and Viral Infections
Source: PLoS One. 2015 Mar 18;10(3):e0120012. doi: 10.1371/journal.pone.0120012 (PMC4364938; doi:10.1371/journal.pone.0120012)
Supplement: S2 Data — (PDF) [file pone.0120012.s002.pdf]

2. Supporting Information S2 - Data analysis and statistical methods

2.1. Measures of accuracy

The signature integrates the levels of three protein biomarkers measured in a subject, and computes a numerical score that reflects the probability of a bacterial infection. To quantify the diagnostic accuracy of the signature we used a cutoff on the score and applied the following measures: Sensitivity, specificity, positive predictive value (PPV), negative predictive value (NPV), total accuracy, positive likelihood ratio (LR+), negative likelihood ratio (LR-), and diagnostic odds ratio (DOR). These measures are defined as follows:

$$Sensitivity = \frac{TP}{TP + FN}$$

$$Specificity = \frac{TN}{TN + FP}$$

$$total\ accuracy = \frac{TP + TN}{TP + FN + TN + FP}$$

$$PPV = \frac{TP}{TP + FP} = \frac{sensitivity \cdot prevalence}{sensitivity \cdot prevalence + (1 - specificity) \cdot (1 - prevalence)}$$

$$NPV = \frac{TN}{TN + FN} = \frac{specificity \cdot (1 - prevalence)}{specificity \cdot (1 - prevalence) + (1 - sensitivity) \cdot (prevalence)}$$

$$LR+ = \frac{Sensitivity}{1 - Specificity}$$

$$LR- = \frac{1 - Sensitivity}{Specificity}$$

$$DOR = \frac{LR+}{LR-}$$

P, N, TP, FP, TN, FN are positives, negatives, true-positives, false-positives, true-negatives, and false-negatives, respectively. Prevalence is the relative frequency of the positive class (i.e., prevalence = P/(P + N)). Unless mentioned otherwise, positives and negatives refer to patients with bacterial and viral infections, respectively.

We also used the area under the receiver operating curve (AUC) to perform cutoff independent comparisons of different diagnostic methods. For details on formulation and confidence interval (CI) computation of the AUC see Hanley and McNeil [1]. We report 95% CIs of the accuracy measures throughout this document.

2.2. Sample size

The primary study objective was to obtain the performance of the signature for classifying patients with viral and bacterial etiologies. We estimate the sample size required to reject the null hypothesis that the sensitivity over the entire population, P, is lower than P_0=75% (H_0: $P \leq P_0$, H_1: $P > P_0$) with significance level of 1% ($\alpha = 0.01$), power of 90% (=1-β) for a difference of 15% ($P_1 - P_0 \geq 0.15$), where P_1 is the empirical sensitivity. This yielded 394 patients (197 viral and 197 bacterial). Additionally we anticipated that roughly 15% of the patients will have an indeterminate source of infection, 10% would be excluded for technical reasons and 10% will be healthy or non-infectious controls. Taken together, the study required the recruitment of at least 607 patients. This requirement was fulfilled because 1002 patients were recruited.

2.3. Building the list of 600 proteins

Systematic literature and bioinformatics screening was applied to identify 600 proteins candidates that had the potential to be differentially expressed in response to infection. The literature review included collection of proteins with a known role in immunity and inflammation both in humans and in animals. The bioinformatics screening included proteins that were differentially expressed on the RNA level across different infections and inflammatory conditions when measured using high throughput sequencing techniques such as microarrays.

2.4. Applying feature selection process to identify the optimal combination of proteins

We applied a feature selection process to identify the optimal combination of proteins. We used two feature selection schemes: mutual-information min-max [2] and forward greedy wrapper [3], which use a series of iterations to add or remove features. The

process was terminated when the increase in performance on the training set was no longer statistically significant (P>0.05). Both processes converged to the same final set of three proteins. To integrate the protein levels into a single score, we examined multiple computational models. Their performances were not significantly different (P>0.1 Supplementary Materials). We chose to a Multinomial Logistic Regression (MLR) model because it provides a probabilistic interpretation by assigning a likelihood score to a patient's diagnosis [4]. The signature uses this property to flag patients whose probability of bacterial infection is intermediate: between 0.35 and 0.55. We use the term 'marginal immune response' to describe these patients because their profile borders between bacterial and viral host-responses.

2.5. Computational model construction

To integrate the protein levels into a single predictive score, we examined multiple computational models including Artificial Neural Networks (ANN), Support Vector Machines (SVM), Bayesian Networks (BN), K-Nearest Neighbor (KNN) and Multinomial Logistic Regression (MLR) [4,5]. The AUCs for distinguishing between bacterial and viral infections obtained on the entire study cohort using a leave-10%-out cross validation were 0.93±0.02 (ANN), 0.93±0.02 (SVM [linear]), 0.94±0.02 [SVM (radial basis function)], 0.92±0.02 (BN), 0.91±0.02 (KNN) and 0.94±0.02 (MLR). We did not observe significant difference in the performances of ANN, SVM and MLR models (P>0.1 when comparing their AUCs). We therefore chose to use MLR because it provides a probabilistic interpretation by assigning a likelihood score to a patient's diagnosis.

We trained and tested the MLR signature for distinguishing between bacterial, viral and non-infectious etiologies. Since the prevalence of underlying etiologies varies across different clinical settings, the model priors were adjusted to reflect a non-bacterial prevalence of 60% and bacterial prevalence of 40%. Within the non-bacterial group the priors were adjusted to 55% viral and 5% non-infectious, to reflect the anticipated higher prevalence of viral versus non-infectious patients among subjects with suspicious for acute infection. The MLR weights and their respective 95% confidence intervals, as well as the p-values associated with each coefficient are summarized in **Tables S1-S2**. In the bacterial versus viral infection analysis the probabilities were adjusted to sum up to 1 ($P_{b\_adjusted}= P_b/[P_b + P_v]$ and $P_{b\_adjusted}= P_v/[P_b + P_v]$, where P_b and P_v correspond to the probability of bacterial and viral infections respectively).

Table S1. MLR coefficients and their respective standard error.

	Class (viral)	Class (bacterial)
**Constant**	-1.299 ± 0.651	-0.378 ± 0.732
**TRAIL**	0.0088 ± 0.0064	-0.020 ± 0.0084
**CRP**	0.0605 ± 0.0145	0.0875 ± 0.015
**IP-10**	0.0053 ± 0.0014	0.0050 ± 0.0014

Table S2. The p-values associated with each MLR coefficient.

	Class (viral)	Class (bacterial)
**Constant**	<0.001	<0.001
**TRAIL**	0.008	<0.001
**CRP**	<0.001	<0.001
**IP-10**	<0.001	<0.001

To further evaluate model calibration and total accuracy we applied the O/E and Brier score (BS) respectively:

$$\frac{O}{E} = \frac{\sum_{i=1}^{N} y_i}{\sum_{i=1}^{N} p_i} = 0.97$$

$$Brier\ Score = \frac{1}{N}\sum_{i=1}^{N}(p_i - y_i)^2 = 0.10$$

where y_i, equals 1 if the ith patient is classified as bacterial and 0 if viral, and $p_i \in [0,1]$ is the predicted probability for a bacterial infection of the ith patient, i=1,…,N. These measures together with the AUCs reported in the main text provide a comprehensive performance evaluation of the model. We also applied these performance measures on the other models reported in the main text. The model distinguishing between bacterial to non-bacterial patients yielded O/E=0.93 and BS=0.09. The model distinguishing between infectious to non-Infectious, patients yielded O/E=0.93 and BS=0.06.

2.6. Logistic calibration curves

In order to assess the validity of the MLR model we compared the estimated predicted probabilities with the actually observed outcomes (Fig. S1). The predicted probabilities are highly compatible with the observed ones, further demonstrating the model validity.

Figure S1. Calibration plot of the MLR model. In the top panel patients were grouped into 10 bins based on their predicted probabilities of a bacterial infection (x-axis), and compared to the observed fraction of bacterial infections within each bin (y-axis). Dashed line is a moving average (of size 5 bins). The bottom panel shows the distribution of predicted probabilities for bacterial (red bars) and viral (blue bars).

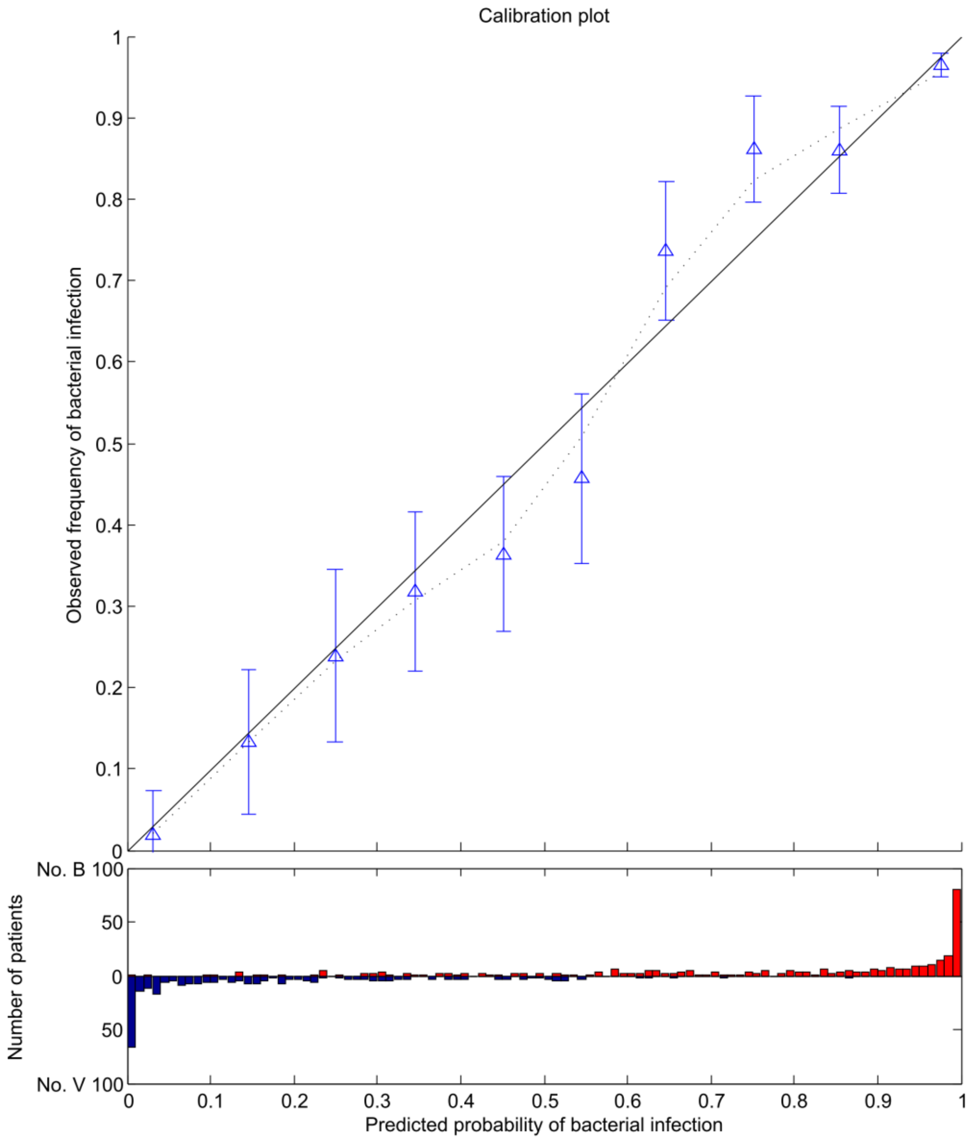

2.7. Using non-linear multinomial logistic models

Non-linear MLR models have the potential to capture more subtle trends in the data compared to linear models, while retaining a probabilistic framework that allows meaningful interpretation of the model results. We examined multiple non-linear MLR models and applied feature selection process to identify the optimal feature combination (Section 2.4). The resulting model is depicted in **Table S3**:

Table S3. Non-linear MLR coefficients

	Class (viral)	Class (bacterial)
**Constant**	-0.8388	5.5123
**CRP**	-0.0487	-0.0636
**CRP^0.5**	1.1367	1.4877
**CRP^2**	-5.14E-05	3.50E-05
**IP-10**	0.0089	0.0085
**TRAIL**	0.0408	0.0646
**TRAIL^0.5**	-0.6064	-1.8039

We examined the model performance on the Microbiologically Confirmed Cohort (AUC of 0.95±0.03), Unanimous Cohort (AUC of 0.95±0.02) and the Study cohort (AUC of 0.93±0.02) **Table S4**. Signature performance improves as the size of the equivocal region increases.

Table S4. Signature measures of accuracy for diagnosing bacterial vs viral infections when using the non-linear MLR model. Performance estimates and their 95% CIs were obtained on the Microbiologically Confirmed Sub-cohort (A; n=241), Unanimous Sub-cohort (B; n=527), and Study Cohort (C; n=653), using different sizes of equivocal regions as indicated. (D-F) Percentage of patients who had equivocal immune response in the Study Cohort when applying different thresholds. (G-H) Signature sensitivity and specificity when applying different equivocal immune response thresholds obtained on the Study Cohort.

A. Microbiologically confirmed sub-cohort (n=241)

Accuracy measure	All patients	Equivocal immune response filter (35-65)	Equivocal immune response filter (30-70)	Equivocal immune response filter (20-80)	Equivocal immune response filter (10-90)
**Total accuracy**	0.89, (0.85, 0.93)	0.93, (0.90, 0.97)	0.94, (0.91, 0.97)	0.96, (0.93, 0.99)	0.98, (0.96, 1.00)
**Sensitivity**	0.88, (0.80, 0.96)	0.93, (0.87, 1.00)	0.95, (0.89, 1.00)	0.96, (0.91, 1.00)	0.96, (0.90, 1.00)
**Specificity**	0.90, (0.87, 0.94)	0.94, (0.90, 0.97)	0.94, (0.90, 0.98)	0.96, (0.93, 0.99)	0.99, (0.97, 1.00)
**% of patients included**	100%	90%	87%	78%	65%

B. Unanimous sub-cohort (n=527)

Accuracy measure	All patients	Equivocal immune response filter (35-65)	Equivocal immune response filter (30-70)	Equivocal immune response filter (20-80)	Equivocal immune response filter (10-90)
**Total accuracy**	0.88, (0.85, 0.91)	0.92, (0.89, 0.94)	0.93, (0.90, 0.95)	0.95, (0.93, 0.97)	0.97, (0.95, 0.99)
**Sensitivity**	0.85, (0.81, 0.89)	0.90, (0.86, 0.94)	0.91, (0.87, 0.95)	0.93, (0.90, 0.97)	0.96, (0.93, 0.99)
**Specificity**	0.91, (0.87, 0.94)	0.93, (0.90, 0.97)	0.94, (0.91, 0.97)	0.96, (0.93, 0.99)	0.98, (0.96, 1.00)
**% of patients included**	100%	90%	86%	76%	63%

C. Entire study cohort (n=653)

Accuracy measure	All patients	Equivocal immune response filter (35-65)	Equivocal immune response filter (30-70)	Equivocal immune response filter (20-80)	Equivocal immune response filter (10-90)
**Total accuracy**	0.85, (0.83, 0.88)	0.89, (0.86, 0.91)	0.90, (0.87, 0.92)	0.92, (0.90, 0.95)	0.95, (0.93, 0.98)
**Sensitivity**	0.83, (0.79, 0.87)	0.87, (0.83, 0.91)	0.89, (0.85, 0.92)	0.92, (0.88, 0.95)	0.95, (0.91, 0.98)
**Specificity**	0.87, (0.84, 0.91)	0.90, (0.87, 0.94)	0.91, (0.88, 0.95)	0.93, (0.89, 0.96)	0.95, (0.92, 0.98)
**% of patients included**	100%	88%	84%	72%	58%

D. Percentage of patients with equivocal immune response using different thresholds

Maximal equivocal immune response threshold (columns); Minimal equivocal immune response threshold (rows)

	0.05	0.1	0.15	0.2	0.25	0.3	0.35	0.4	0.45	0.5	0.55	0.6	0.65	0.7	0.75	0.8	0.85	0.9	0.95
0.05		6.6%	10.4%	13.9%	17.6%	20.5%	22.7%	24.0%	26.3%	29.1%	31.2%	33.2%	34.8%	36.3%	38.6%	40.9%	44.0%	47.2%	52.8%
0.1			3.8%	7.4%	11.0%	13.9%	16.1%	17.5%	19.8%	22.5%	24.7%	26.6%	28.2%	29.7%	32.0%	34.3%	37.4%	40.6%	46.2%
0.15				3.5%	7.2%	10.1%	12.3%	13.6%	15.9%	18.7%	20.8%	22.8%	24.3%	25.9%	28.2%	30.5%	33.5%	36.8%	42.4%
0.2					3.7%	6.6%	8.7%	10.1%	12.4%	15.2%	17.3%	19.3%	20.8%	22.4%	24.7%	27.0%	30.0%	33.2%	38.9%
0.25						2.9%	5.1%	6.4%	8.7%	11.5%	13.6%	15.6%	17.2%	18.7%	21.0%	23.3%	26.3%	29.6%	35.2%
0.3							2.1%	3.5%	5.8%	8.6%	10.7%	12.7%	14.2%	15.8%	18.1%	20.4%	23.4%	26.6%	32.3%
0.35								1.4%	3.7%	6.4%	8.6%	10.6%	12.1%	13.6%	15.9%	18.2%	21.3%	24.5%	30.2%
0.4									2.3%	5.1%	7.2%	9.2%	10.7%	12.3%	14.5%	16.8%	19.9%	23.1%	28.8%
0.45										2.8%	4.9%	6.9%	8.4%	10.0%	12.3%	14.5%	17.6%	20.8%	26.5%
0.5											2.1%	4.1%	5.7%	7.2%	9.5%	11.8%	14.9%	18.1%	23.7%
0.55												2.0%	3.5%	5.1%	7.4%	9.6%	12.7%	15.9%	21.6%
0.6													1.5%	3.1%	5.4%	7.7%	10.7%	13.9%	19.6%
0.65														1.5%	3.8%	6.1%	9.2%	12.4%	18.1%
0.7															2.3%	4.6%	7.7%	10.9%	16.5%
0.75																2.3%	5.4%	8.6%	14.2%
0.8																	3.1%	6.3%	11.9%
0.85																		3.2%	8.9%
0.9																			5.7%
0.95																			

E. Percentage of bacterial patients with equivocal immune response using different thresholds

Maximal equivocal immune response threshold (columns); Minimal equivocal immune response threshold (rows)

	0.05	0.1	0.15	0.2	0.25	0.3	0.35	0.4	0.45	0.5	0.55	0.6	0.65	0.7	0.75	0.8	0.85	0.9	0.95
0.05		2.5%	3.4%	5.3%	7.5%	9.1%	10.3%	11.3%	13.8%	16.6%	18.8%	21.6%	23.5%	26.0%	29.5%	33.5%	38.2%	43.6%	53.6%
0.1			0.9%	2.8%	5.0%	6.6%	7.8%	8.8%	11.3%	14.1%	16.3%	19.1%	21.0%	23.5%	27.0%	31.0%	35.7%	41.1%	51.1%
0.15				1.9%	4.1%	5.6%	6.9%	7.8%	10.3%	13.2%	15.4%	18.2%	20.1%	22.6%	26.0%	30.1%	34.8%	40.1%	50.2%
0.2					2.2%	3.8%	5.0%	6.0%	8.5%	11.3%	13.5%	16.3%	18.2%	20.7%	24.1%	28.2%	32.9%	38.2%	48.3%
0.25						1.6%	2.8%	3.8%	6.3%	9.1%	11.3%	14.1%	16.0%	18.5%	21.9%	26.0%	30.7%	36.1%	46.1%
0.3							1.3%	2.2%	4.7%	7.5%	9.7%	12.5%	14.4%	16.9%	20.4%	24.5%	29.2%	34.5%	44.5%
0.35								0.9%	3.4%	6.3%	8.5%	11.3%	13.2%	15.7%	19.1%	23.2%	27.9%	33.2%	43.3%
0.4									2.5%	5.3%	7.5%	10.3%	12.2%	14.7%	18.2%	22.3%	27.0%	32.3%	42.3%
0.45										2.8%	5.0%	7.8%	9.7%	12.2%	15.7%	19.7%	24.5%	29.8%	39.8%
0.5											2.2%	5.0%	6.9%	9.4%	12.9%	16.9%	21.6%	27.0%	37.0%
0.55												2.8%	4.7%	7.2%	10.7%	14.7%	19.4%	24.8%	34.8%
0.6													1.9%	4.4%	7.8%	11.9%	16.6%	21.9%	32.0%
0.65														2.5%	6.0%	10.0%	14.7%	20.1%	30.1%
0.7															3.4%	7.5%	12.2%	17.6%	27.6%
0.75																4.1%	8.8%	14.1%	24.1%
0.8																	4.7%	10.0%	20.1%
0.85																		5.3%	15.4%
0.9																			10.0%
0.95																			

F. Percentage of viral patients with equivocal immune response using different thresholds

Maximal equivocal immune response threshold

Minimal equivocal immune response threshold

	0.05	0.1	0.15	0.2	0.25	0.3	0.35	0.4	0.45	0.5	0.55	0.6	0.65	0.7	0.75	0.8	0.85	0.9	0.95
0.05		10.5%	17.1%	22.2%	27.2%	31.4%	34.4%	36.2%	38.3%	41.0%	43.1%	44.3%	45.5%	46.1%	47.3%	47.9%	49.4%	50.6%	52.1%
0.1			6.6%	11.7%	16.8%	21.0%	24.0%	25.7%	27.8%	30.5%	32.6%	33.8%	35.0%	35.6%	36.8%	37.4%	38.9%	40.1%	41.6%
0.15				5.1%	10.2%	14.4%	17.4%	19.2%	21.3%	24.0%	26.0%	27.2%	28.4%	29.0%	30.2%	30.8%	32.3%	33.5%	35.0%
0.2					5.1%	9.3%	12.3%	14.1%	16.2%	18.9%	21.0%	22.2%	23.4%	24.0%	25.1%	25.7%	27.2%	28.4%	29.9%
0.25						4.2%	7.2%	9.0%	11.1%	13.8%	15.9%	17.1%	18.3%	18.9%	20.1%	20.7%	22.2%	23.4%	24.9%
0.3							3.0%	4.8%	6.9%	9.6%	11.7%	12.9%	14.1%	14.7%	15.9%	16.5%	18.0%	19.2%	20.7%
0.35								1.8%	3.9%	6.6%	8.7%	9.9%	11.1%	11.7%	12.9%	13.5%	15.0%	16.2%	17.7%
0.4									2.1%	4.8%	6.9%	8.1%	9.3%	9.9%	11.1%	11.7%	13.2%	14.4%	15.9%
0.45										2.7%	4.8%	6.0%	7.2%	7.8%	9.0%	9.6%	11.1%	12.3%	13.8%
0.5											2.1%	3.3%	4.5%	5.1%	6.3%	6.9%	8.4%	9.6%	11.1%
0.55												1.2%	2.4%	3.0%	4.2%	4.8%	6.3%	7.5%	9.0%
0.6													1.2%	1.8%	3.0%	3.6%	5.1%	6.3%	7.8%
0.65														0.6%	1.8%	2.4%	3.9%	5.1%	6.6%
0.7															1.2%	1.8%	3.3%	4.5%	6.0%
0.75																0.6%	2.1%	3.3%	4.8%
0.8																	1.5%	2.7%	4.2%
0.85																		1.2%	2.7%
0.9																			1.5%
0.95																			

G. Signature sensitivity calculated for different equivocal immune response thresholds

Maximal equivocal immune response threshold

Minimal equivocal immune response threshold

	0.05	0.1	0.15	0.2	0.25	0.3	0.35	0.4	0.45	0.5	0.55	0.6	0.65	0.7	0.75	0.8	0.85	0.9	0.95
0.05		84.6%	85.4%	87.1%	89.2%	90.7%	92.0%	92.9%	95.6%	98.9%	98.8%	98.8%	98.8%	98.7%	98.7%	98.6%	98.5%	98.3%	98.0%
0.1			83.2%	84.8%	86.8%	88.3%	89.5%	90.4%	92.9%	96.0%	95.9%	95.7%	95.6%	95.5%	95.3%	95.0%	94.6%	94.1%	92.9%
0.15				84.0%	85.9%	87.4%	88.6%	89.5%	92.0%	94.9%	94.8%	94.6%	94.5%	94.3%	94.1%	93.7%	93.3%	92.7%	91.2%
0.2					84.3%	85.7%	86.8%	87.7%	90.1%	92.9%	92.8%	92.5%	92.3%	92.1%	91.7%	91.3%	90.7%	89.8%	87.9%
0.25						83.8%	84.8%	85.7%	88.0%	90.7%	90.5%	90.1%	89.9%	89.6%	89.2%	88.6%	87.8%	86.8%	84.3%
0.3							83.5%	84.3%	86.5%	89.2%	88.9%	88.5%	88.3%	87.9%	87.4%	86.7%	85.8%	84.7%	81.9%
0.35								83.2%	85.4%	88.0%	87.7%	87.3%	87.0%	86.6%	86.0%	85.3%	84.3%	83.1%	80.1%
0.4									84.6%	87.1%	86.8%	86.4%	86.1%	85.7%	85.1%	84.3%	83.3%	81.9%	78.8%
0.45										84.8%	84.5%	84.0%	83.7%	83.2%	82.5%	81.6%	80.5%	79.0%	75.5%
0.5											82.1%	81.5%	81.1%	80.6%	79.9%	78.9%	77.6%	76.0%	72.1%
0.55												81.9%	81.6%	81.1%	80.4%	79.4%	78.2%	76.7%	73.1%
0.6													82.1%	81.6%	81.0%	80.1%	78.9%	77.5%	74.2%
0.65														82.0%	81.3%	80.5%	79.4%	78.0%	74.9%
0.7															81.8%	81.0%	80.0%	78.7%	75.8%
0.75																81.7%	80.8%	79.6%	76.9%
0.8																	81.6%	80.5%	78.0%
0.85																		81.5%	79.3%
0.9																			80.5%
0.95																			

H. Signature specificity calculated for different equivocal immune response thresholds

Maximal equivocal immune response threshold

Minimal equivocal immune response threshold	0.05	0.1	0.15	0.2	0.25	0.3	0.35	0.4	0.45	0.5	0.55	0.6	0.65	0.7	0.75	0.8	0.85	0.9	0.95
0.05		86.3%	85.2%	84.2%	83.1%	82.1%	81.3%	80.8%	80.1%	79.2%	82.1%	83.9%	85.7%	86.7%	88.6%	89.7%	92.3%	94.5%	97.5%
0.1			86.9%	86.1%	85.3%	84.5%	83.9%	83.5%	83.0%	82.3%	84.9%	86.4%	88.0%	88.8%	90.5%	91.4%	93.6%	95.5%	97.9%
0.15				87.1%	86.3%	85.7%	85.1%	84.8%	84.4%	83.9%	86.2%	87.7%	89.1%	89.9%	91.4%	92.2%	94.2%	95.9%	98.2%
0.2					87.1%	86.5%	86.0%	85.7%	85.4%	84.9%	87.1%	88.5%	89.8%	90.6%	92.0%	92.7%	94.7%	96.2%	98.3%
0.25						87.2%	86.8%	86.5%	86.2%	85.8%	87.9%	89.2%	90.5%	91.1%	92.5%	93.2%	95.0%	96.5%	98.4%
0.3							87.3%	87.1%	86.8%	86.4%	88.5%	89.7%	90.9%	91.6%	92.9%	93.5%	95.3%	96.7%	98.5%
0.35								87.5%	87.2%	86.9%	88.9%	90.0%	91.2%	91.9%	93.1%	93.8%	95.4%	96.8%	98.5%
0.4									87.5%	87.1%	89.1%	90.2%	91.4%	92.0%	93.3%	93.9%	95.5%	96.9%	98.6%
0.45										87.4%	89.3%	90.4%	91.6%	92.2%	93.4%	94.0%	95.6%	96.9%	98.6%
0.5											89.6%	90.7%	91.8%	92.4%	93.6%	94.2%	95.8%	97.0%	98.7%
0.55												88.8%	89.9%	90.4%	91.6%	92.1%	93.6%	94.8%	96.4%
0.6													88.8%	89.3%	90.4%	91.0%	92.4%	93.6%	95.1%
0.65														88.3%	89.3%	89.9%	91.3%	92.4%	93.9%
0.7															88.8%	89.3%	90.7%	91.8%	93.3%
0.75																88.3%	89.6%	90.7%	92.1%
0.8																	89.1%	90.2%	91.6%
0.85																		88.8%	90.2%
0.9																			89.1%
0.95																			

Signature performance was further examined on the Study Cohort when excluding the following two subgroups: (i) patients whose blood sample was taken after more than 3 days of antibiotic treatment in the hospital and (ii) patients with a suspected gastroenteritis. The first group is likely to include patients at convalescence, which are responding to treatment and therefore their immune response at time of sampling is already non-bacterial. The second group showed slightly reduced performance (Figure 3) compared to other infection types. Details of the model performance on the Microbiologically Confirmed Cohort (AUC of 0.96±0.04), Unanimous Cohort (AUC of 0.96±0.02) and the Study Cohort (AUC of 0.95±0.02) are further depicted in **Table S5**.

Table S5. Signature measures of accuracy for diagnosing bacterial vs viral infections using the non-linear MLR model. Performance estimates and their 95% CIs were obtained on the Microbiologically Confirmed Sub-cohort (A; n=200), Unanimous Sub-cohort (B; n=402), and Study Cohort (C; n=491), when excluding patients with over 3 days of antibiotics treatment at the hospital and/or suspicion of gastroenteritis.

A. Microbiologically confirmed sub-cohort (n=200)

Accuracy measure	All patients	Equivocal immune response filter (35-65)	Equivocal immune response filter (30-70)	Equivocal immune response filter (20-80)	Equivocal immune response filter (10-90)
Total accuracy	0.91, (0.86, 0.95)	0.95, (0.92, 1)	0.95, (0.92, 0.99)	0.96, (0.93, 0.99)	0.98, (0.96, 1)
Sensitivity	0.90, (0.82, 0.99)	0.96, (0.89, 1)	0.96, (0.89, 1)	0.95, (0.89, 1)	0.94, (87, 1)
Specificity	0.91, (0.86, 0.95)	0.95, (0.91, 0.99)	0.95, (0.92, 0.99)	0.97, (0.93, 1)	1, (1,1)
% of patients included	100%	90%	88%	80%	65%

B. Unanimous sub-cohort (n=402)

Accuracy measure	All patients	Equivocal immune response filter (35-65)	Equivocal immune response filter (30-70)	Equivocal immune response filter (20-80)	Equivocal immune response filter (10-90)
Total accuracy	0.91, (0.88, 0.94)	0.94, (0.92, 0.97)	0.95, (0.93, 0.97)	0.96, (0.94, 0.98)	0.98, (0.96, 1)
Sensitivity	0.89, (0.85, 0.94)	0.93, (0.89, 0.97)	0.94, (0.90, 0.98)	0.95, (0.92, 0.99)	0.98, (0.95,1)
Specificity	0.92, (0.88, 0.96)	0.95, (0.92, 0.98)	0.95, (0.93, 0.98)	0.97, (0.94, 0.99)	0.99, (0.97, 1)
% of patients included	100%	91%	88%	79%	65%

C. Entire study cohort (n=491)

Accuracy measure	All patients	Equivocal immune response filter (35-65)	Equivocal immune response filter (30-70)	Equivocal immune response filter (20-80)	Equivocal immune response filter (10-90)
Total accuracy	0.88, (0.85, 0.91)	0.91, (0.89, 0.94)	0.93, (0.90, 0.95)	0.94, (0.92, 0.97)	0.97, (0.95, 0.99)
Sensitivity	0.87, (0.83, 0.92)	0.91, (0.87, 0.95)	0.92, (0.88, 0.96)	0.95, (0.91, 0.98)	0.97, (0.94, 1)
Specificity	0.89, (0.85, 0.92)	0.92, (0.89, 0.96)	0.93, (0.90, 0.96)	0.94, (0.91, 0.97)	0.97, (0.94, 1)
% of patients included	100%	88%	85%	74%	59%

References

1. Hanley JA, McNeil BJ (1983) A method of comparing the areas under receiver operating characteristic curves derived from the same cases. Radiology 148: 839–843.

2. Torkkola MK, Torkkola K, Guyon I, Elisseeff A (2003) Journal of Machine Learning Research 3 (2003) 1415-1438 Submitted 5/02; Published 3/03 Feature Extraction by Non-Parametric Mutual Information. Journal of Machine Learning Research 3: 1415–1438.

3. Kohavi R, John GH (1997) Wrappers for feature subset selection. Artificial Intelligence 97: 273–324. doi:10.1016/S0004-3702(97)00043-X.

4. Jr DWH, Lemeshow S (2004) Applied Logistic Regression. John Wiley & Sons. 397 p.

5. Duda RO, Hart PE, Stork DG (2000) Pattern Classification (2nd Edition). Wiley-Interscience.
