# Supplementary material for: A Novel Host-Proteome Signature for Distinguishing between Acute Bacterial and Viral Infections
Source: PLoS One. 2015 Mar 18;10(3):e0120012. doi: 10.1371/journal.pone.0120012 (PMC4364938; doi:10.1371/journal.pone.0120012)
Supplement: S3 Data — (PDF) [file pone.0120012.s003.pdf]

3. Supporting Information S3 - Cohort characteristics

3.1. Summary of the patient cohorts used in this study

A total of 1002 patients were recruited and 892 were enrolled (110 were excluded based on pre-determined exclusion criteria). Based on the reference standard process described in the 'Methods' section, patients were assigned to four different diagnosis groups: (i) bacterial; (ii) viral; (iii) no apparent infectious disease or healthy (controls); and (iv) indeterminate. Patients diagnosed by the panel as having mixed infections (bacteria plus virus) were labeled as bacterial because they are managed similarly (e.g. treated with antibiotics) (see 'Methods', **Fig. 1A**). In total, 89% of all enrolled patients were assigned a diagnosis, a rate which approaches the literature-documented limit [1–3]. The following sections provide a detailed description of patient characteristics, which includes all the patients with a final diagnosis (n=794): 765 patients included in the study cohort and 29 patients for which the serum samples were depleted during the screening phase (**Fig. 1**).

3.2. Age and gender distribution

Patients of all ages were recruited to the study. The patients with agreed diagnosis (diagnosed patients; n=794) included more pediatric (≤18 years) than adult (>18 years) patients (445 patients [56%] vs 349 [44%]). The age distribution was relatively uniform for patients aged 20-80 years and peaked at <4 years of age for pediatric patients (**Fig. S2**). The observed age distribution for pediatric patients is consistent with that expected and represents the background distribution in the inpatient setting [4] (e.g., the emergency department [ED], pediatrics departments, and internal departments). Patients of both genders were recruited to the study. The patient population was balanced in respect to gender distribution (47% females, 53% males).

Figure S2. Age distribution of the diagnosed patients. A. The entire study population (n=794); B. Pediatric patients only (n=445).

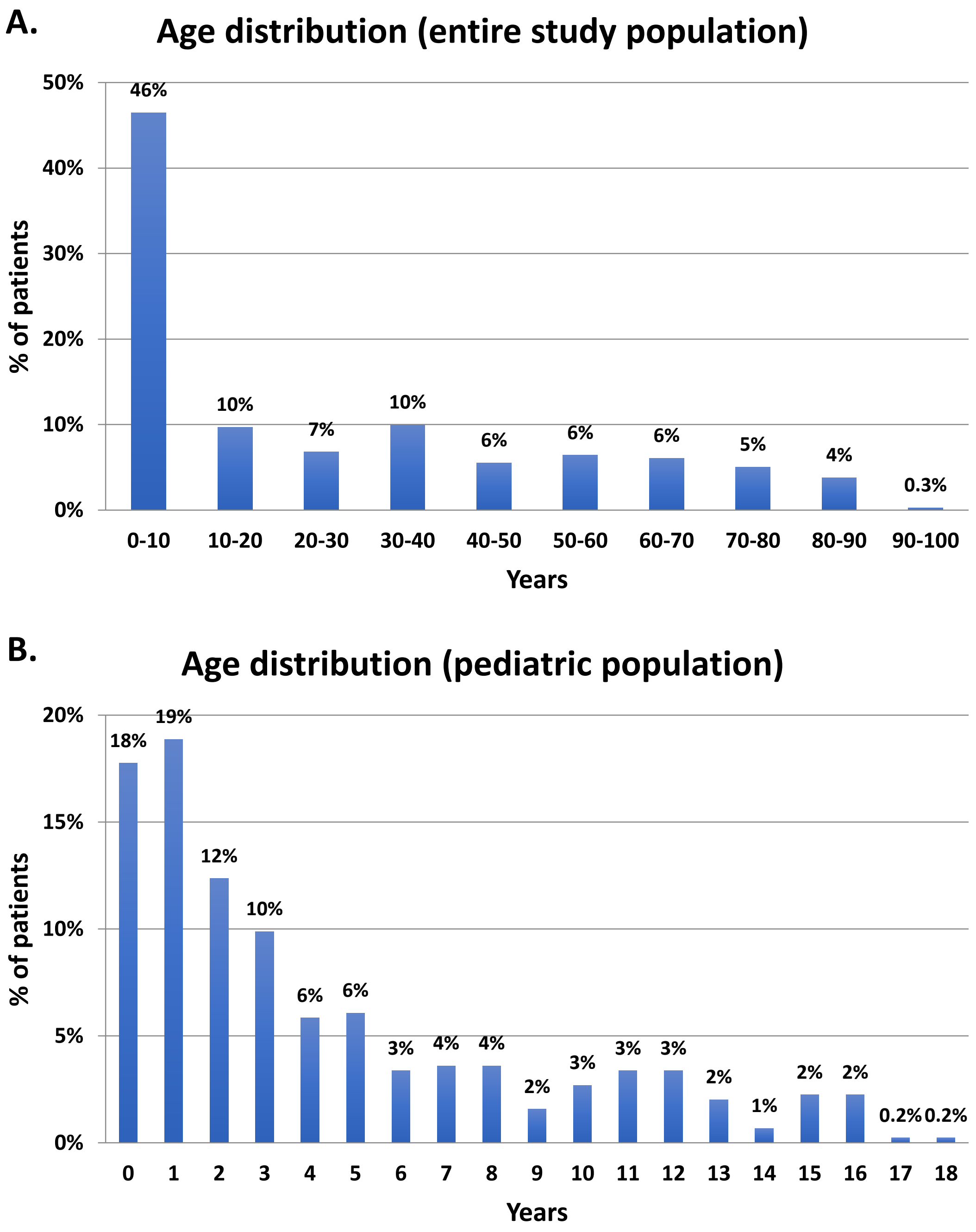

3.3. Detected pathogens

We used a wide panel of microbiological tools in order to maximize pathogen detection rate. At least one pathogen was detected in 65% of patients with an acute infectious disease (56% of all 794 diagnosed patients). A total of 36 different pathogens were actively detected using multiplex PCR, antigen detection, and serological investigation. Additional 20 pathogens were detected using standard culture techniques or in-house PCR. Altogether, 56 different pathogens from all major pathogenic subgroups were detected (**Fig. S3A**). This rate of pathogen identification is similar to that reported in previously published studies [5–9] and included pathogens from all major pathogenic subgroups (Gram-negative bacteria, Gram-positive bacteria, atypical bacteria, RNA viruses, and DNA viruses). In 13% of the patients, pathogens from more than one of the aforementioned pathogenic subgroups were detected (**Fig. S3A**).

The pathogenic strains found in this study are responsible for the vast majority of acute infectious diseases in the Western world and included key pathogens such as influenza A/B, respiratory syncytial virus (RSV), parainfluenza, *E. Coli*, Group A *Streptococcus*, etc. Notably, analysis of the detected pathogens revealed that none of the pathogens is dominant (**Fig. S3B**).

Figure S3. Distribution of detected pathogens in diagnosed patients (n=794). A. Distribution of detected pathogens by pathogenic subgroups; B. Distribution of detected pathogens by strain (strains detected from >1% of patients are presented). Distribution represents % of positive detections in patients with diagnosed infectious disease.

A.

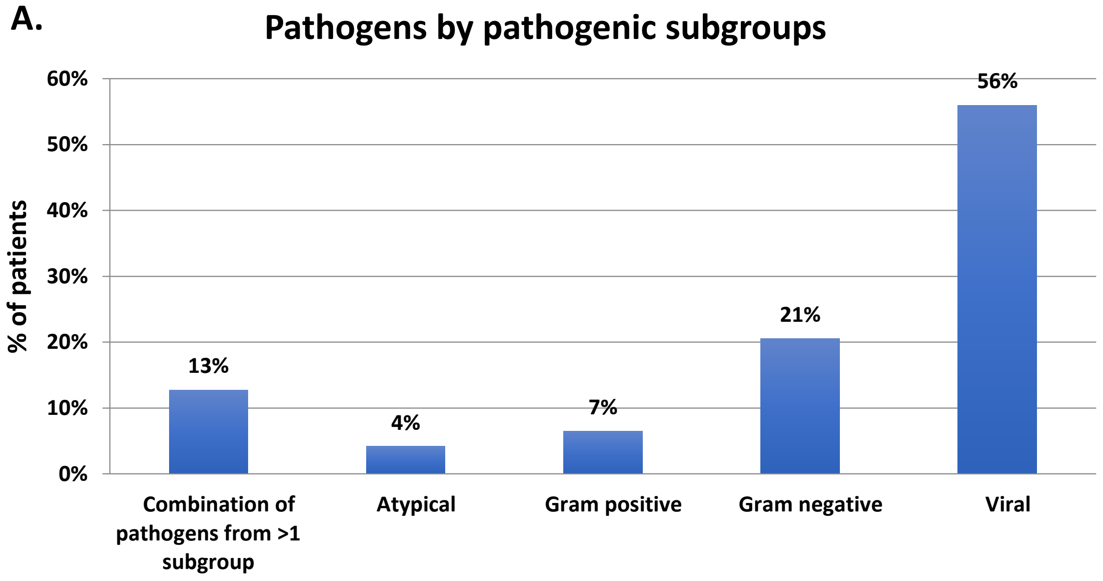

B.

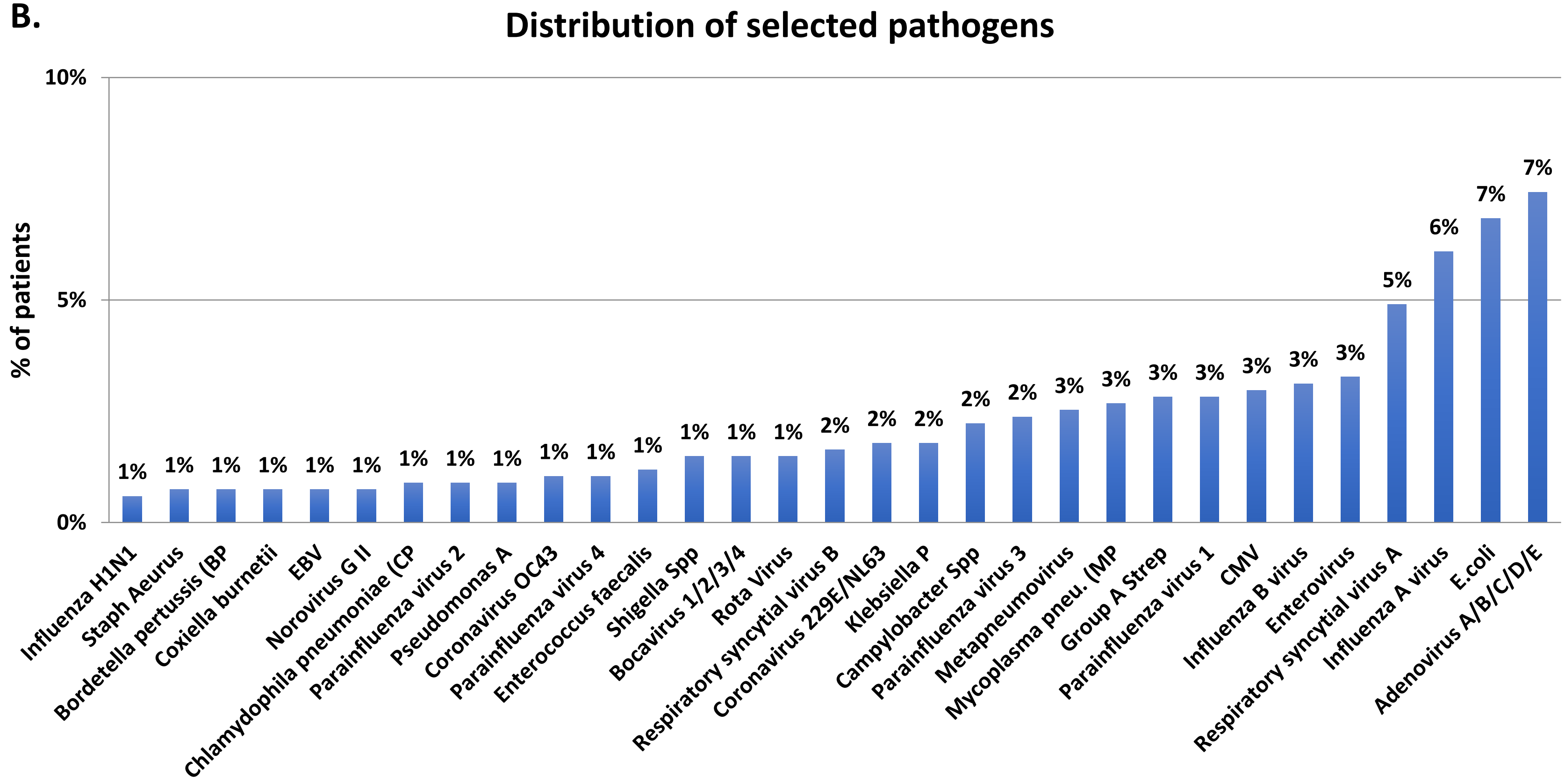
Distribution of selected pathogens
% of patients
10%
5%
0%
1%
1%
1%
1%
1%
1%
1%
1%
1%
1%
1%
1%
1%
1%
1%
2%
2%
2%
2%
2%
3%
3%
3%
3%
3%
3%
3%
5%
6%
7%
7%
Influenza H1N1
Staph Aeurus
Bordetella pertussis (BP
Coxiella burnetii
EBV
Norovirus G II
Chlamydophila pneumoniae (CP
Parainfluenza virus 2
Pseudomonas A
Coronavirus OC43
Parainfluenza virus 4
Enterococcus faecalis
Shigella Spp
Bocavirus 1/2/3/4
Rota Virus
Respiratory syncytial virus B
Coronavirus 229E/NL63
Klebsiella P
Campylobacter Spp
Parainfluenza virus 3
Metapneumovirus
Mycoplasma pneu. (MP
Group A Strep
Parainfluenza virus 1
CMV
Influenza B virus
Enterovirus
Respiratory syncytial virus A
Influenza A virus
E.coli
Adenovirus A/B/C/D/E

3.4. Involved physiologic systems and clinical syndromes

The infectious disease patients (all diagnosed patients [n=794], excluding those with non-infectious diseases or healthy subjects, n=673) presented with infections in a variety of physiologic systems (Fig. S4). The most frequently involved physiologic system was the respiratory system (46%), followed by systemic infections (22%). All infections that did not involve the aforementioned systems and were not gastrointestinal, urinary, cardiovascular, or central nervous system (CNS) infections were categorized as 'Other' (e.g., cellulitis, abscess). The observed distribution of physiologic system involvement represents the natural distribution and is consistent with that reported for large cohorts of patients sampled year-round [10].

Figure S4. Distribution of involved physiologic systems in patients diagnosed with an infectious disease (n=673).

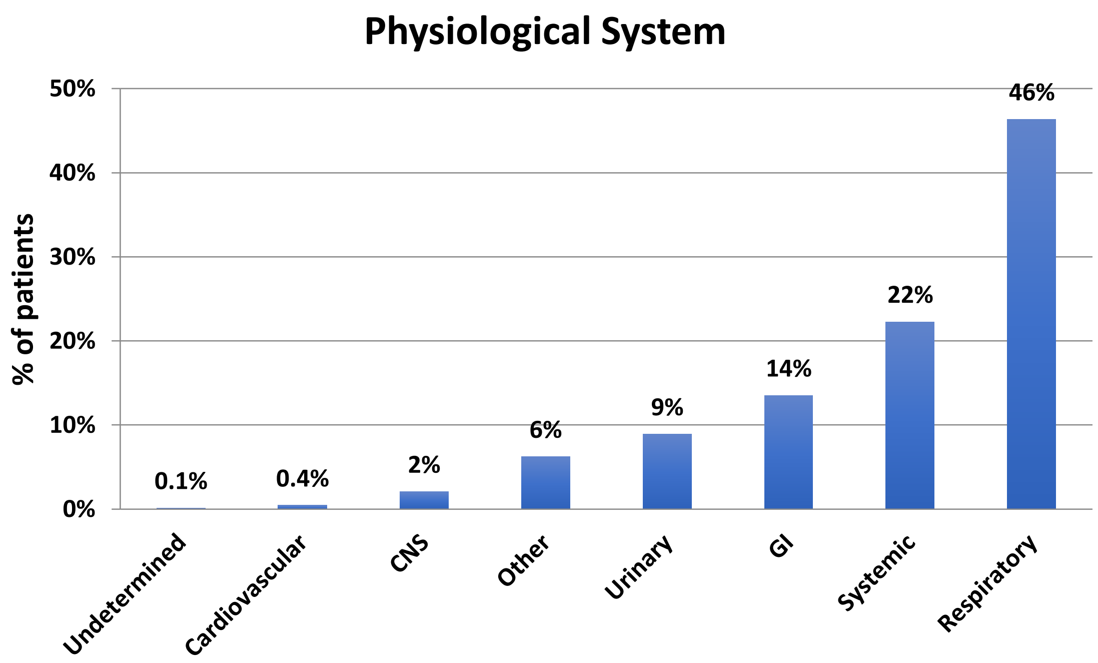

The diagnosed patients in our study (n=794) presented with a variety of clinical syndromes (Fig. S5) that reflects the expected clinical heterogeneity in a cohort of pediatric and adult patients collected year-round. The most frequent clinical syndrome was LRTI (21%) including mainly pneumonia, bronchitis, bronchiolitis, chronic obstructive pulmonary disease (COPD) exacerbation, and non-specific LRTI. The second most frequent syndrome was systemic infection (19%) including mainly fever without a source and occult bacteremia cases. Systemic infections were primarily detected in

children <3 years of age but were also detected in a few adult patients. Systemic infections constitute a real clinical challenge as balancing between patient risk and the costs of testing/treatment is unclear. The third most frequent clinical syndrome was URTI (19%) including mainly acute tonsillitis, acute pharyngitis, non-specific URTI, acute sinusitis, and acute otitis media. The next most frequent syndromes were gastroenteritis (12%), UTI (7%), and cellulitis (4%). CNS infections (2%) included septic and aseptic meningitis. Additional clinical syndromes (1%) were classified as 'Other' and included less common infections (e.g., otitis externa, epididymitis, etc.). The observed pattern of clinical syndrome distribution represents most of the frequent and clinically relevant syndromes and is consistent with previously published large studies [4].

Figure S5. Distribution of clinical syndromes (all diagnosed patients, n=794). A. Major clinical syndromes; B. Specific clinical syndromes.

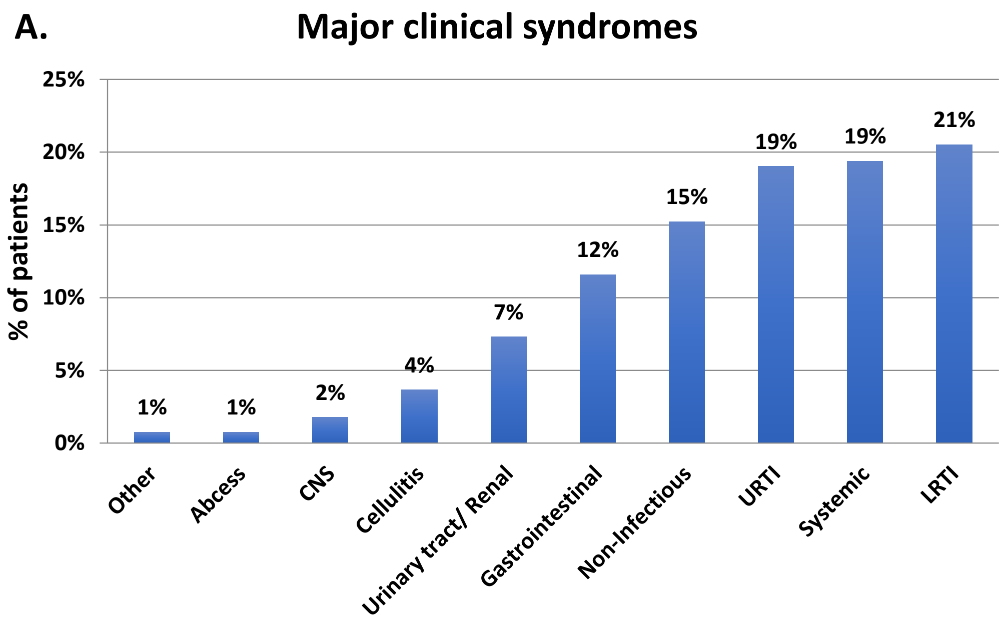

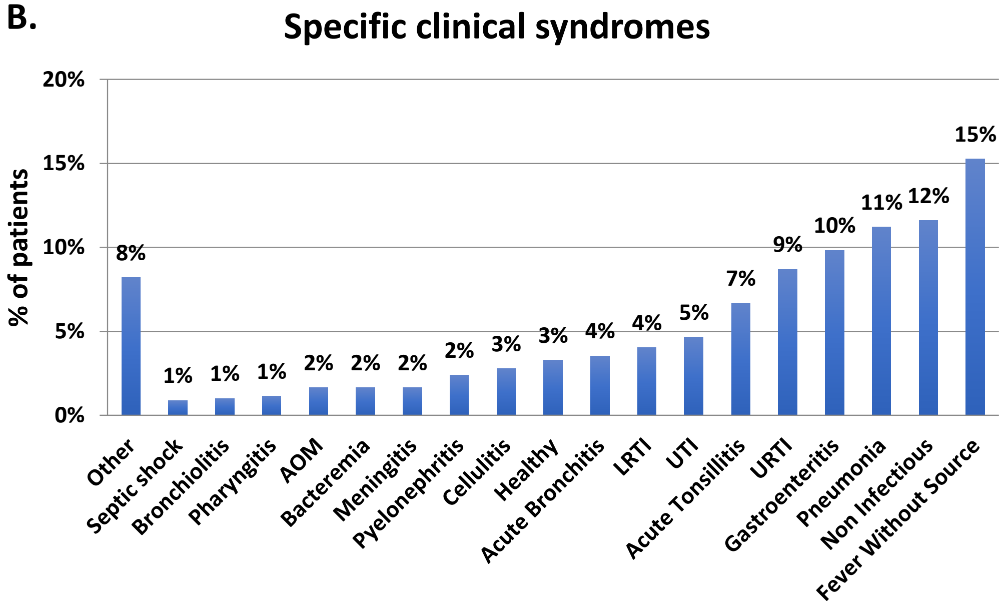

3.5. Core body temperature

Core body temperature is an important parameter in evaluating infectious disease severity. We examined the distribution of maximal body temperatures in all of the diagnosed patients (n=794) using the highest measured body temperature (per-os or per-rectum). The distribution of the maximal body temperatures was relatively uniform between 38°C and 40°C with a peak of at 39°C (Fig. S6). Body temperature <37.5°C was reported for 15% of patients (the subgroup of patients with non-infectious diseases or healthy subjects). Body temperature ≥40.5°C was rare (<3% of patients). Altogether, the observed distribution represents the normal range of temperatures in the clinical setting [4].

Figure S6. Distribution of maximal body temperatures (n=794).

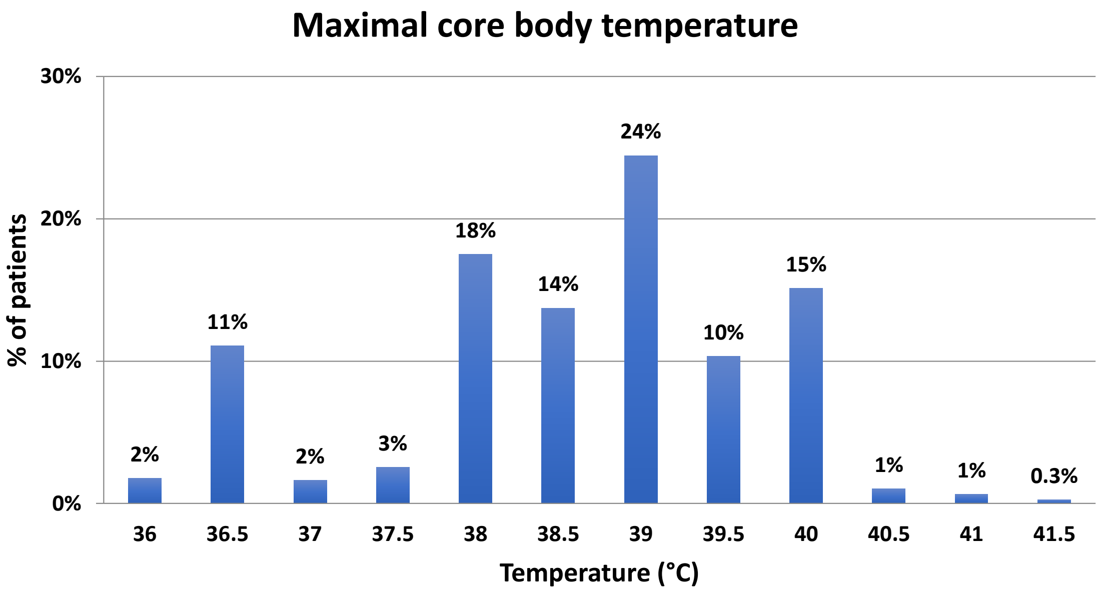

3.6. Time from symptom onset

'Time from symptom onset' was defined as the duration (days) from the appearance of the *first* presenting symptom (the first presenting symptom could be fever but could also be another symptom such as nausea or headache preceding the fever) till the time of blood sample collection. The distribution of 'time from symptom onset' in our cohort (all diagnosed patients, n=794) peaked at 2-4 days after the initiation of symptoms (35% of patients) with substantial proportions of patients turning to medical assistance either sooner or later (Fig. S7).

Figure S7. Distribution of time from initiation of symptoms (n=794). N/A – healthy controls or patients for which data was not obtained.

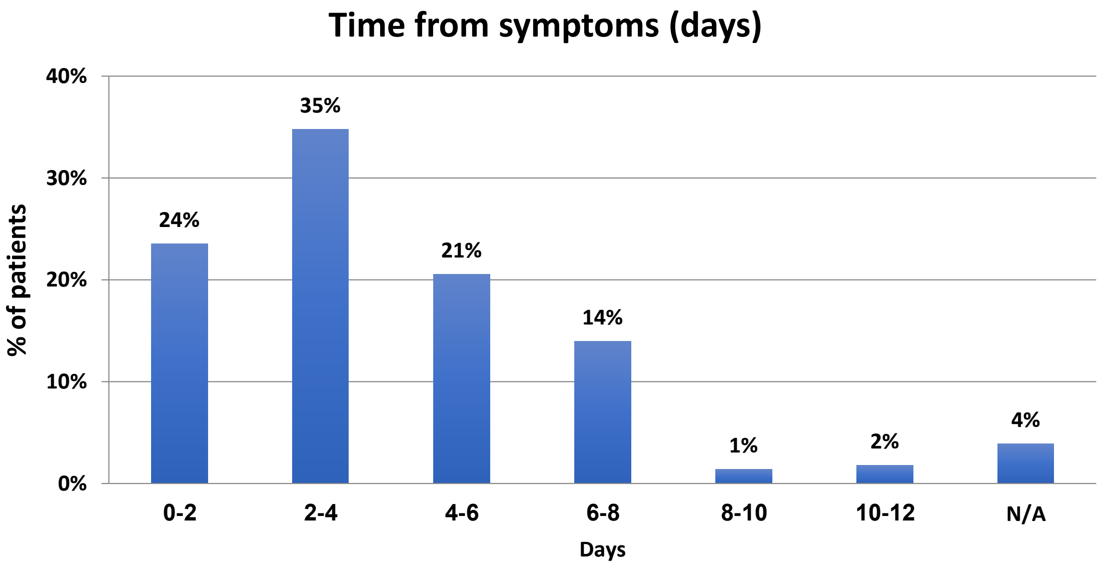

3.7. Comorbidities and chronic drug regimens

Comorbidities and chronic drug regimens may, theoretically, affect a diagnostic test. Out of the diagnosed patients 62% had no comorbidities whereas 38% had ≥1 chronic disease. In addition, 75% of patients were not treated with chronic medications and 25% were treated with ≥1 chronic medication. The most frequent chronic diseases in our patient population were hypertension, hyperlipidemia, lung diseases (e.g., COPD, asthma, etc.), diabetes mellitus (mostly type 2), and ischemic heart disease, mirroring the most common chronic diseases in the Western world (Fig. S8A). The distribution of chronic drugs used by our patient population strongly correlated with the range of reported chronic diseases (e.g., 29% of the patients with comorbidities had hyperlipidemia and lipid lowering agents were the most frequently used drugs). Other

frequently used drugs included aspirin, blood glucose control drugs, and beta blockers (**Fig. S8B**).

Figure S8. Comorbidities-related characterization of the patient population. A. Distribution of comorbidities (all chronically ill patients, n=305); B. Distribution of chronic medications (all chronically ill patients, n=305). Of note, some of the patients presented with several chronic diseases, and treated with several chronic medications.

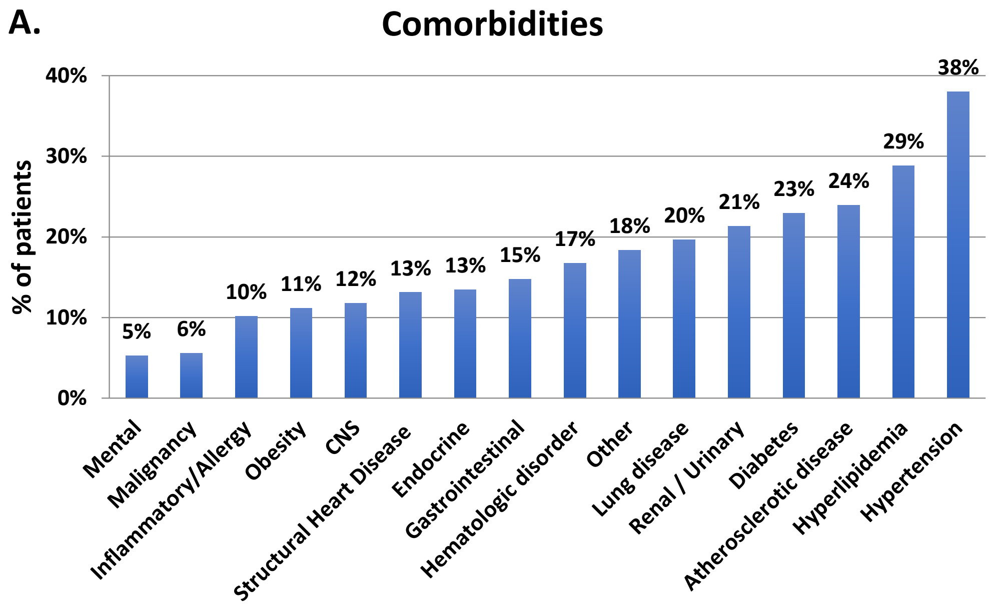

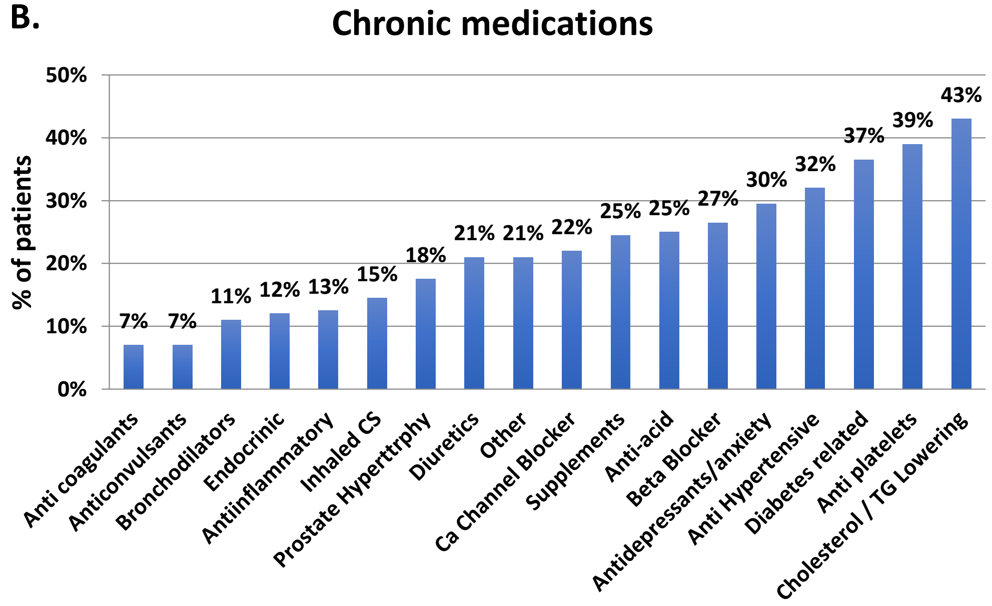

3.8. Patient recruitment sites

Pediatric patients (≤18 years) were recruited from pediatric emergency departments (PED), pediatric wards and surgical departments, and adults (>18 years) from emergency departments (ED), internal medicine departments and surgical departments. The pediatric ED was the most common recruitment site (39%) and the other sites were comparable (17-20%) reflecting a relatively balanced recruitment process. The ratio between ED patients and hospitalized patients was ~1:1 for adults and ~2:1 for children (Fig. S9).

Figure S9. Distribution of recruitment sites (diagnosed patients, n=794).

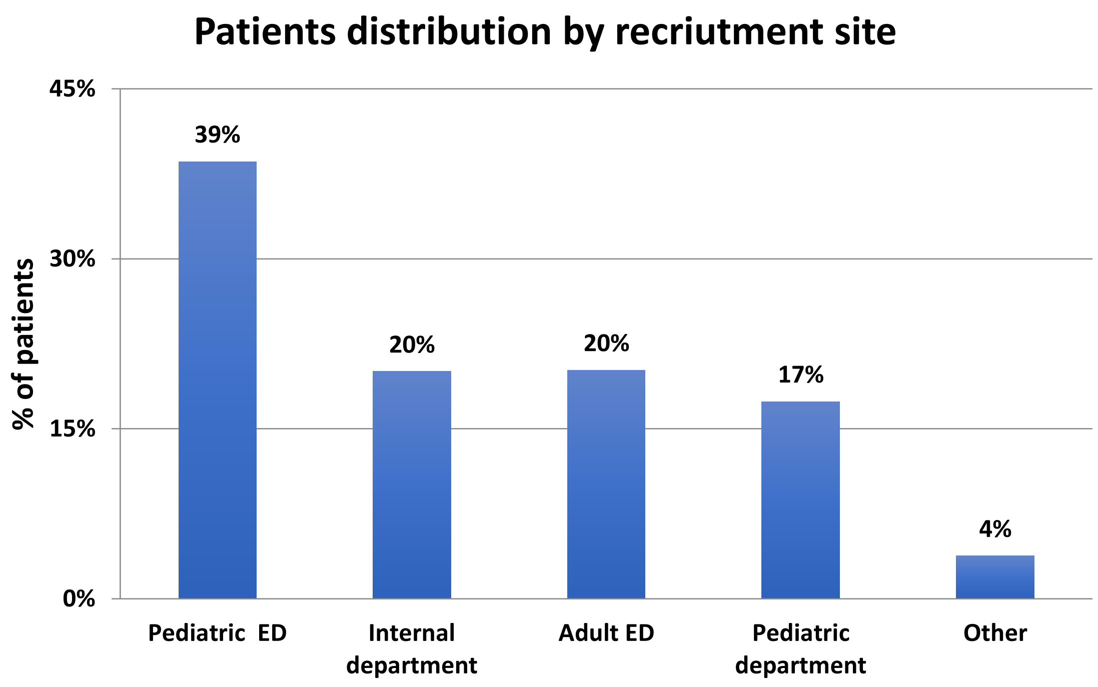

3.9. Characteristics of excluded patients

Of the 1002 patients recruited for the study, 110 patients (11%) were excluded (some patients fulfilled more than one exclusion criterion). The most frequent reason for exclusion was having a fever below the study threshold of 37.5°C (n=54), followed by time from symptom initiation of >12 days (n=26) and having a recent (in the preceding 14 days) infectious disease (n=22). Other reasons for exclusion included having an active malignancy (n=14), and being immunocompromised (e.g., due to treatment with an immunosuppressive drug; n=2).

3.10. Characteristics of indeterminate patients

A total of 98 patients were defined as indeterminate based on the inability of the expert panel to reliably establish a final diagnosis, despite the rigorous collection of laboratory and clinical information. While it is not possible to directly examine the signature performance in these patients in the absence of a reference standard, it is possible to analyze their host-protein response in order to assess whether they differ from patients with a reference standard. We compared the distribution of TRAIL, IP-10 and CRP in acute infection patients with a reference standard (n=653) to those without a reference standard (n=98). No statistically significant difference was observed (Kolmogorov Smirnov test P = 0.20, 0.25, 0.46 for TRAIL, IP-10 and CRP, respectively). The similarity in the host-protein response between patients with and without a reference standard implies that the present approach may be useful for diagnosing indeterminate patients in the clinical setting.

References

1. Clements H, Stephenson T, Gabriel V, Harrison T, Millar M, et al. (2000) Rationalised Prescribing for Community Acquired Pneumonia: A Closed Loop Audit. Arch Dis Child 83: 320–324. doi:10.1136/adc.83.4.320.

2. Johnstone J, Majumdar SR, Fox JD, Marrie TJ (2008) Viral Infection in Adults Hospitalized With Community-Acquired Pneumonia Prevalence, Pathogens, and Presentation. Chest 134: 1141–1148. doi:10.1378/chest.08-0888.

3. Hatipoğlu N, Somer A, Badur S, Unüvar E, Akçay-Ciblak M, et al. (2011) Viral etiology in hospitalized children with acute lower respiratory tract infection. Turk J Pediatr 53: 508–516.

4. Craig JC, Williams GJ, Jones M, Codarini M, Macaskill P, et al. (2010) The accuracy of clinical symptoms and signs for the diagnosis of serious bacterial infection in young febrile children: prospective cohort study of 15 781 febrile illnesses. BMJ 340. doi:10.1136/bmj.c1594.

5. Restrepo MI, Mortensen EM, Velez JA, Frei C, Anzueto A (2008) A Comparative Study of Community-Acquired Pneumonia Patients Admitted to the Ward and the ICU*. Chest 133: 610–617. doi:10.1378/chest.07-1456.

6. Song J-H, Oh WS, Kang C-I, Chung DR, Peck KR, et al. (2008) Epidemiology and clinical outcomes of community-acquired pneumonia in adult patients in Asian countries: a prospective study by the Asian network for surveillance of resistant pathogens. International Journal of Antimicrobial Agents 31: 107–114. doi:10.1016/j.ijantimicag.2007.09.014.

7. Johansson N, Kalin M, Tiveljung-Lindell A, Giske CG, Hedlund J (2010) Etiology of community-acquired pneumonia: increased microbiological yield with new diagnostic methods. Clin Infect Dis 50: 202–209. doi:10.1086/648678.

8. Shibli F, Chazan B, Nitzan O, Flatau E, Edelstein H, et al. (2010) Etiology of community-acquired pneumonia in hospitalized patients in northern Israel. Isr Med Assoc J 12: 477–482.

9. Cillóniz C, Ewig S, Polverino E, Marcos MA, Esquinas C, et al. (2011) Microbial Aetiology of Community-Acquired Pneumonia and Its Relation to Severity. Thorax 66: 340–346. doi:10.1136/thx.2010.143982.

10. NAMCS/NHAMCS - NCHS Reports Using Ambulatory Health Care Data (n.d.). Available: http://www.cdc.gov/nchs/ahcd/ahcd_reports.htm. Accessed 17 January 2013.
