# Supplementary material for: A Novel Host-Proteome Signature for Distinguishing between Acute Bacterial and Viral Infections
Source: PLoS One. 2015 Mar 18;10(3):e0120012. doi: 10.1371/journal.pone.0120012 (PMC4364938; doi:10.1371/journal.pone.0120012)
Supplement: S4 Data — (PDF) [file pone.0120012.s004.pdf]

4. Supporting Information S4 - The signature performance remains robust across different patient subgroups

We demonstrated that the signature remained robust across a wide range of patient characteristics including age, clinical syndrome, time from symptom onset, maximal temperature, pathogen species, comorbidities, and the clinical site with AUCs ranging from 0.87 to 1.0 (Fig. 4). In this section, we review the performance of the signature across additional patient subgroups.

4.1. Stratification by chronic drug regimens

In real-world clinical practice, patients are often under various chronic drug regimens, which could, potentially, affect the level of proteins comprising the signature. We therefore examined whether the most used drugs (by categories) in our cohort impact the signature's performance. None of the evaluated drug groups were associated with significant alterations in the signature's accuracy (Table S6).

Table S6. Evaluation of the signature's sensitivity to various types of chronic drug regimens.

Drug category	AUC [95% CI]		Total patients, n	Bacterial patients, n	Viral patients, n
Anti Hypertensive	0.95	[0.90, 1.00]	50	43	7
Anti platelets	0.99	[0.96, 1.00]	54	48	6
Anti-acid	0.90	[0.80, 1.00]	42	35	7
Antidepressants	0.98	[0.93, 1.00]	29	25	4
Beta Blocker	0.95	[0.88, 1.00]	40	35	5
Ca Channel Blocker	0.94	[0.86, 1.00]	39	34	5
Cholesterol / TG Lowering	0.94	[0.89, 1.00]	64	53	11
Diabetic	0.87	[0.74, 1.00]	40	35	5
Diuretics	0.93	[0.83, 1.00]	30	25	5
Hormonal	0.98	[0.93, 1.00]	18	14	4
Inhaled CS	0.95	[0.87, 0.99]	26	18	8
Prostate Hypertrophy	0.94	[0.84, 1.00]	25	21	4

4.2. Sepsis based stratification

Sepsis is a potentially fatal medical condition characterized by a whole-body inflammatory state (called systemic inflammatory response syndrome [SIRS]) and the

presence of a known or suspected infection [1]. Patients with a bacterial sepsis benefit from early antibiotic therapy; delayed or misdiagnosis can have serious or even fatal consequences [2,3]. We focused on adult patients for whom the definition of SIRS is clear and examined the ability of the signature to distinguish between adult patients with bacterial sepsis and those with viral infections as well as between adult patients with bacterial sepsis and those with viral sepsis.

Adult patients with bacterial sepsis were defined according to the American College of Chest Physicians and the Society of Critical Care Medicine [2]. SIRS was defined by the presence of at least two of the following findings: (i) body temperature <36°C or >38°C, (ii) heart rate >90 beats per minute, (iii) respiratory rate >20 breaths per minute or, on blood gas, a $PaCO_2$ <32 mm Hg (4.3 kPa), and (iv) WBC <4,000 cells/mm^3 or >12,000 cells/mm^3 or >10% band forms. We found that the signature achieved very high levels of accuracy in distinguishing between adult patients with bacterial sepsis and those with viral sepsis (AUC of 0.97 and 0.93 for the Unanimous sub-cohort and the entire study cohort, respectively). These results demonstrate the utility of the signature in differentiating adult patients with bacterial sepsis from adult patients with viral infections.

Table S7. Signature accuracy in diagnosing bacterial sepsis vs viral sepsis in adult patients.

	AUC [95% CI]		Total patients, n	Bacterial patients, n	Viral patients, n
Unanimous sub-cohort	0.97	[0.94, 1.00]	114	93	21
Study cohort	0.93	[0.89, 0.97]	147	112	35

4.3. Bacterial vs non-bacterial patients stratification

Antibiotic misuse typically stems from the use of these drugs to treat non-bacterial (viral or non-infectious) patients or due to delayed or missed diagnosis of bacterial infections. Therefore, we further examined the signature performance for distinguishing between bacterial and non-bacterial patients. The entire study cohort was evaluated using leave-10%-out cross-validation, yielding AUC of 0.94±0.02. Improved performances were shown when evaluating the Unanimous sub-cohort (AUC of 0.96±0.02), and after filtering out patients with a marginal immune response (**Table S8**).

Table S8. Signature measures of accuracy for diagnosing bacterial vs non-bacterial (viral and non-infectious) patients. A. Performance estimates and their 95% CIs were obtained using a leave-10%-out cross-validation using the entire study cohort ($n_{Bacterial}$=319, $n_{Non\text{-}bacterial}$=446) and the Unanimous sub-cohort ($n_{Bacterial}$=256, $n_{Non\text{-}bacterial}$=383). B. The analysis was repeated after filtering out patients with a marginal immune response (entire study cohort [$n_{Bacterial}$=292, $n_{Non\text{-}bacterial}$=387, $n_{Marginal}$=86] and Unanimous sub-cohort [$n_{Bacterial}$=237, $n_{Non\text{-}bacterial}$=343, $n_{Marginal}$=59]), which resembles the way clinicians are likely to use the signature.

	A. All patients		B. Marginal immune response filter	
**Accuracy measure**	**Study cohort**	**Unanimous sub-cohort**	**Study cohort**	**Unanimous sub-cohort**
**AUC**	0.94 (0.92, 0.96)	0.96 (0.94, 0.98)	0.95 (0.93, 0.97)	0.96 (0.94, 0.98)
**Total accuracy**	0.88 (0.85, 0.91)	0.91 (0.89, 0.93)	0.91 (0.89, 0.93)	0.93 (0.91, 0.95)
**Sensitivity**	0.87 (0.83, 0.91)	0.88 (0.85, 0.91)	0.91 (0.88, 0.95)	0.92 (0.88, 0.95)
**Specificity**	0.90 (0.87, 0.93)	0.93 (0.91, 0.95)	0.92 (0.89, 0.95)	0.94 (0.91, 0.96)
**LR+**	8.7 (6, 12)	12.6 (9, 18)	11.4 (8, 16)	15.3 (10, 23)
**LR-**	0.14 (0.11, 0.19)	0.13 (0.09, 0.18)	0.1 (0.07, 0.14)	0.08 (0.05, 0.13)
**DOR**	60 (38, 94)	97 (56, 168)	116 (67, 200)	180 (94, 344)

4.4. Technically excluded patients

We also tested the signature on the subgroup of patients who were technically excluded, but had unanimous labeling by the expert panel, which yielded an AUC of 0.96±0.06 ($n_{Bacterial}$=27, n_{Viral}=14). This might suggest that the signature is applicable more broadly to conditions that were excluded in the present study (e.g. sub-febrile patients).

4.5. Antibiotics based stratification

Of the 653 patients with suspicion of acute infection, 427 received antibiotics (299 had bacterial diagnosis and 128 had viral diagnosis). The AUC of the signature for distinguishing between the bacterial and viral infected patients in the antibiotics treated patients sub-cohort was 0.93±0.02. We did not observe a statistically significant difference between the performance on the antibiotics treated patients and the general cohort (0.94±0.02 vs 0.93±0.02; P = 0.5).

References

1. Levy MM, Fink MP, Marshall JC, Abraham E, Angus D, et al. (2003) 2001 SCCM/ESICM/ACCP/ATS/SIS International Sepsis Definitions Conference. Crit Care Med 31: 1250–1256. doi:10.1097/01.CCM.0000050454.01978.3B.

2. Bone RC, Balk RA, Cerra FB, Dellinger RP, Fein AM, et al. (1992) Definitions for sepsis and organ failure and guidelines for the use of innovative therapies in sepsis. The ACCP/SCCM Consensus Conference Committee. American College of Chest Physicians/Society of Critical Care Medicine. Chest 101: 1644–1655.

3. Rivers E, Nguyen B, Havstad S, Ressler J, Muzzin A, et al. (2001) Early goal-directed therapy in the treatment of severe sepsis and septic shock. N Engl J Med 345: 1368–1377. doi:10.1056/NEJMoa010307.
