# Supplementary material for: A Novel Host-Proteome Signature for Distinguishing between Acute Bacterial and Viral Infections
Source: PLoS One. 2015 Mar 18;10(3):e0120012. doi: 10.1371/journal.pone.0120012 (PMC4364938; doi:10.1371/journal.pone.0120012)
Supplement: S5 Data — (PDF) [file pone.0120012.s005.pdf]

5. Supporting Information S5 - Performance analysis as a function of the prevalence of bacterial infections

The prevalence of bacterial and viral infections is setting dependent. For example, in the winter, a pediatrician in the outpatient setting is expected to encounter substantially more viral infections than a physician in the hospital internal department during the summer. Notably, some measures of diagnostic accuracy such as AUC, sensitivity, and specificity are invariant to the underlying prevalence, whereas other measures of accuracy, such as PPV and NPV are prevalence dependent. In this section, we review the expected signature performance in terms of PPV and NPV in clinical settings with different prevalence of bacterial and viral infections.

As the basis for this analysis we used the signature accuracy measures obtained using the entire study cohort (bacterial, viral) and the Unanimous sub-cohort (bacterial, viral) after filtering out patients with a marginal immune response. The prevalence of bacterial infections in the Unanimous sub-cohort was 51.7% yielding a PPV of 93% ± 3% and NPV of 93% ± 3%. The prevalence of bacterial infections in the entire study cohort was 48.7% yielding a PPV of 89% ± 3% and NPV of 92% ± 3%.

We used the measured sensitivity and specificity to compute the expected changes in the signature PPV and NPV as a function of the prevalence of bacterial infections (**Fig. S10**).

Figure S10. Extrapolated PPV and NPV values for the signature as a function of the prevalence of bacterial infections, A. Unanimous sub-cohort (bacterial, viral; n=527), B. The entire study cohort (bacterial, viral; n=653).

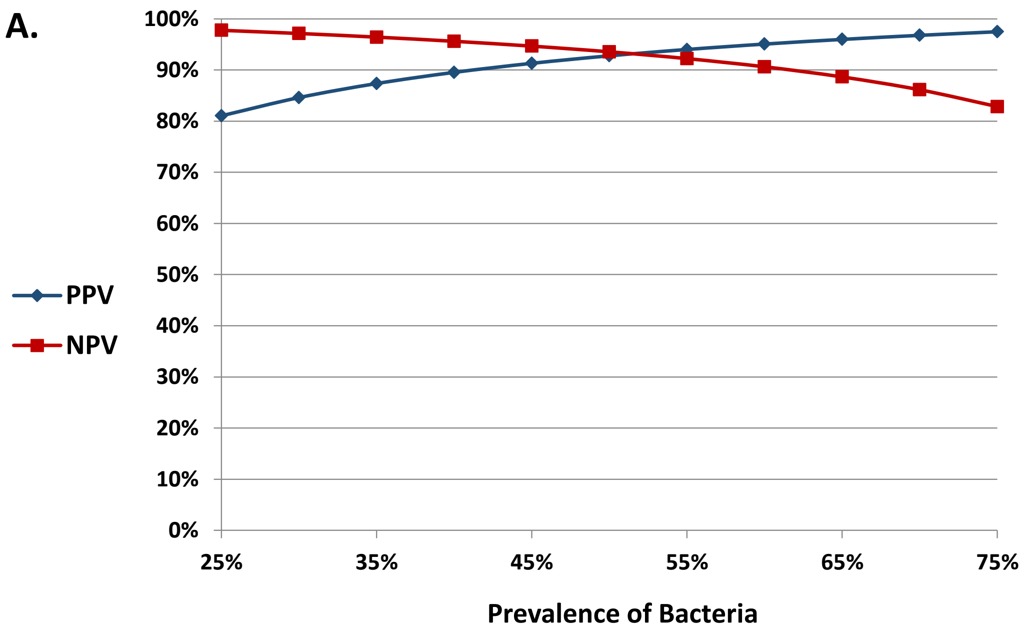

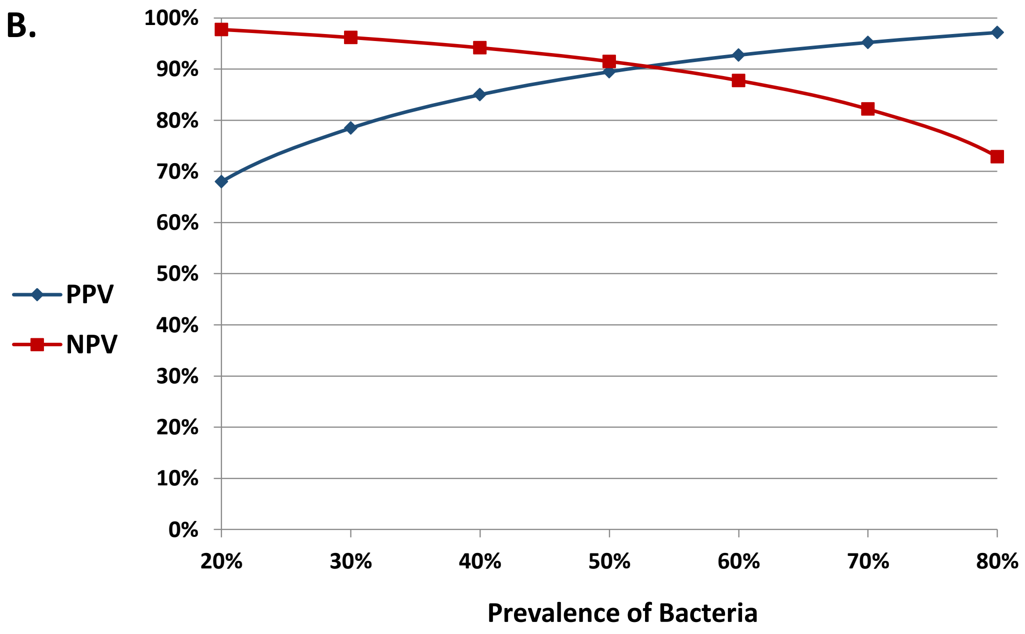

Examples of different clinical settings and the extrapolated signature PPV and NPV for each of them are presented in Table S9.

Table S9. Extrapolated signature PPV and NPV in different clinical settings, based on the Unanimous sub-cohort.

Setting	Age	Prevalence of Bacterial infections*	PPV	NPV
Outpatient	Children	20%	76%	98%
Outpatient	Adults	35%	85%	97%
Inpatient	Children	50%	93%	94%
Inpatient	Adults	80%	98%	78%

*An average annual prevalence. Estimates of bacterial infection prevalence are based on data reported in the Bacterial etiology chapter, Part 7 of Harrison's Internal Medicine 17th Edition [1].

References

1. Thorn GW, Adams, Braunwald, Isselbacher, Petersdorf (1977) Harrison's Principles of Internal Medicine. 8th Edition.
