# Supplementary material for: A Novel Host-Proteome Signature for Distinguishing between Acute Bacterial and Viral Infections
Source: PLoS One. 2015 Mar 18;10(3):e0120012. doi: 10.1371/journal.pone.0120012 (PMC4364938; doi:10.1371/journal.pone.0120012)
Supplement: S6 Data — (PDF) [file pone.0120012.s006.pdf]

6. Supporting Information S6 - Comparison of the signature with alternative diagnostic methods

6.1. The signature outperforms standard laboratory and clinical parameters for diagnosing bacterial vs viral infections

Standard laboratory and clinical parameters, some of which are routinely used in clinical practice to aid in the differential diagnosis of an infection source, were evaluated in the entire study cohort (bacterial, viral, non-infectious, n=765). The evaluated parameters included ANC, % neutrophils, % lymphocytes, WBC, and maximal temperature. In accordance with the well-established clinical role of these parameters, we observed a statistically significant difference in their levels between bacterial and viral patients (Fig. S11). For example, bacterial patients had increased levels of ANC (t-test $P < 10^{-24}$), and WBC (t-test $P < 10^{-10}$), whereas viral patients had a higher % lymphocytes (t-test $P < 10^{-31}$). The signature was significantly more accurate than any of the individual features (t-test $P<10^{-18}$) and their combinations (t-test $P<10^{-15}$), see Fig. 3A).

Figure S11. Box plots of clinical parameters and laboratory measurements in bacterial, viral, and non-infectious patients (as indicated) in the entire study cohort (bacterial, viral, non-infectious; n=765). Red line and circle correspond to group median and average respectively. T-test p-values between bacterial and viral groups and between infectious (bacterial and viral) vs non-infectious (including healthy subjects) are depicted.

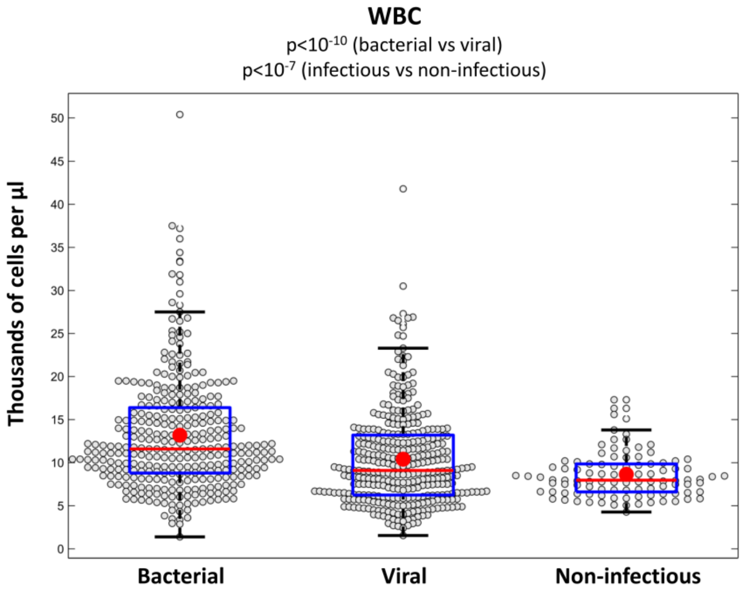

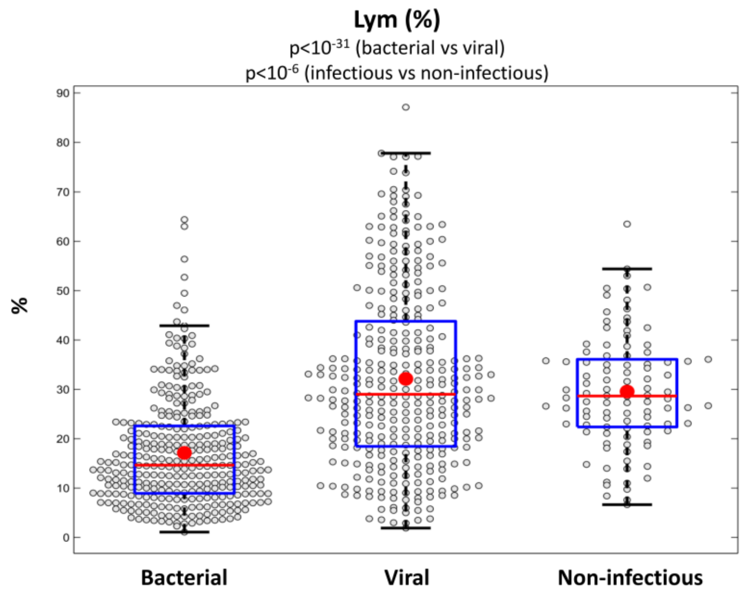
Lym (%)
p<10^-31 (bacterial vs viral)
p<10^-6 (infectious vs non-infectious)
%
0
10
20
30
40
50
60
70
80
90
Bacterial
Viral
Non-infectious

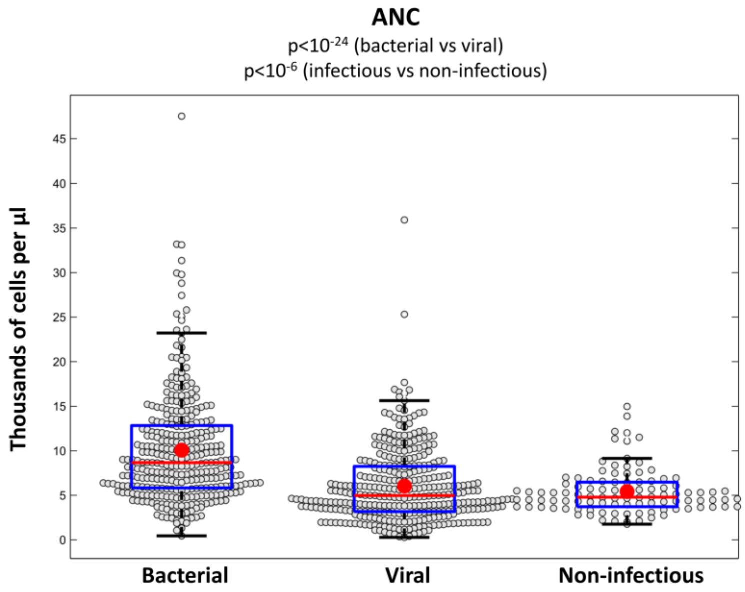
ANC
p<10^-24 (bacterial vs viral)
p<10^-6 (infectious vs non-infectious)
Thousands of cells per µl
0
5
10
15
20
25
30
35
40
45
Bacterial
Viral
Non-infectious

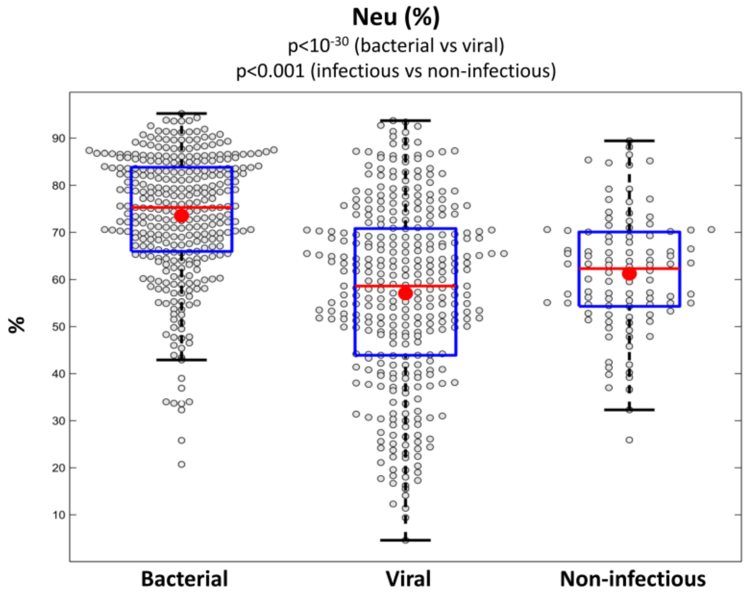

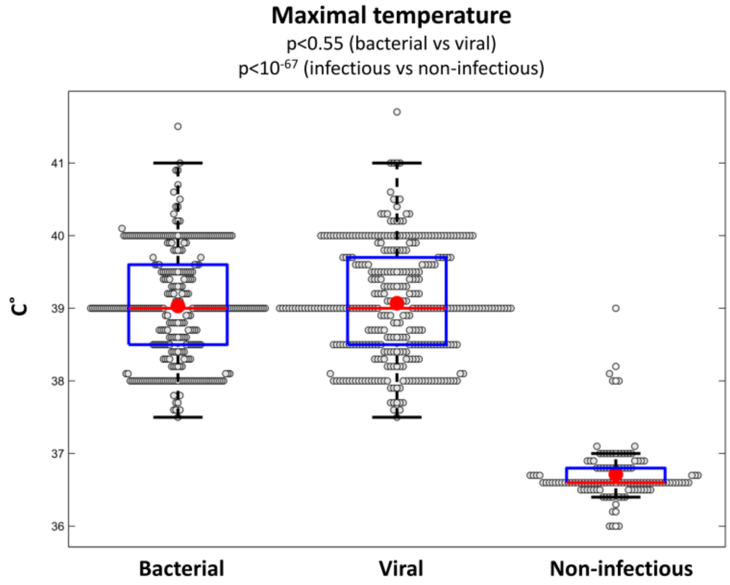

6.2. The signature outperforms protein biomarkers with a well-established immunological role

The signature outperformed all clinical parameters and the 600 proteins that were evaluated during the screening phase (see Fig. 3). The following section further compares the signature to selected proteins that are routinely used in the clinical setting or that have an immunological role.

One of the most widely used and useful protein biomarkers for differentiating sepsis from other non-infectious causes of SIRS in critically ill patients is procalcitonin (PCT) [1].

Whether PCT can be used to distinguish between local bacterial and viral infections is less clear. To test this, we measured PCT concentrations in 101 randomly selected patients from the study cohort ($n_{Bacterial}$=51, n_{Viral}=50) and 76 randomly selected patients from the Unanimous sub-cohort ($n_{Bacterial}$=39, n_{Viral}=37) and compared the diagnostic accuracy based on PCT levels to that of the signature. PCT accuracy was calculated using the standard cutoffs routinely applied in the clinical setting (0.1 ng/mL, 0.25 ng/mL, 0.5 ng/mL, and 1 ng/mL [2–6]. Maximal PCT sensitivity of 69% was attained at a cutoff of 0.1mg/mL and resulted in a specificity of 62% (for the Unanimous sub-cohort [bacterial, viral]). For the same cohort, the signature showed significantly higher sensitivity of 94% ($P < 0.001$) and specificity of 93% ($P < 0.001$) (**Fig. S12A**). A comparison using the patients from the entire study cohort (bacterial, viral) showed similar results (**Fig. S12B**).

Overall, despite its high diagnostic and prognostic value for sepsis detection in critically ill patients, our results indicate that PCT is less accurate in distinguishing between patients with local infections (bacterial vs viral).

Figure S12. Comparison of the performance of the signature and PCT using different cutoffs. A. Performance measured in 76 patients from the Unanimous sub-cohort (bacterial, viral); B. Performance measured in 101 patients from the entire study cohort (bacterial, viral). Error bars represent 95% CI. Signature sensitivity and specificity were calculated after filtering out 14% of the patients with a marginal immune response.

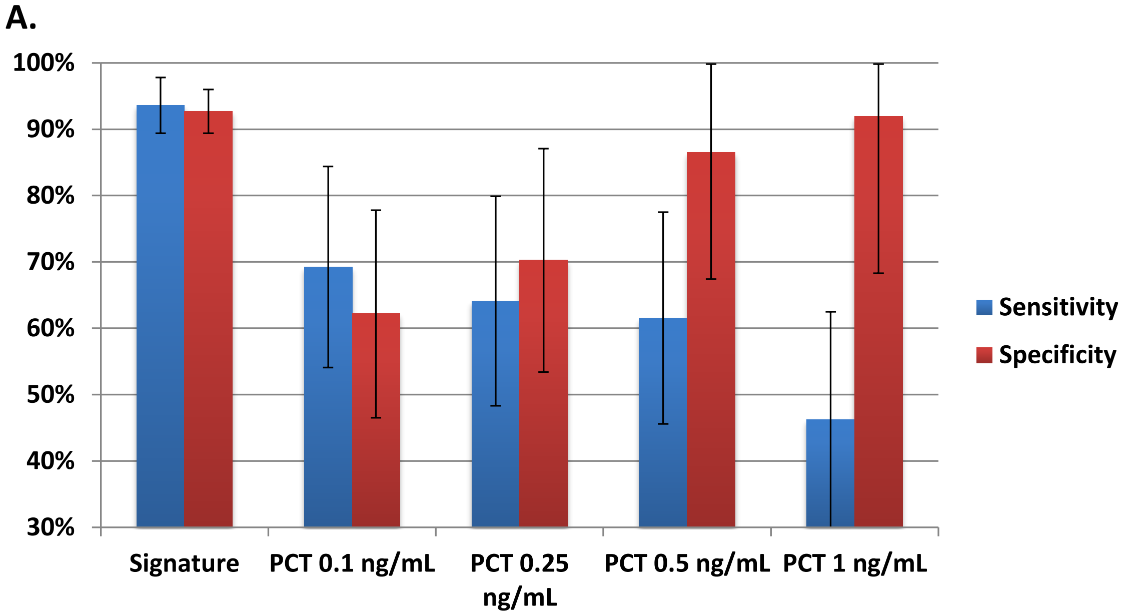

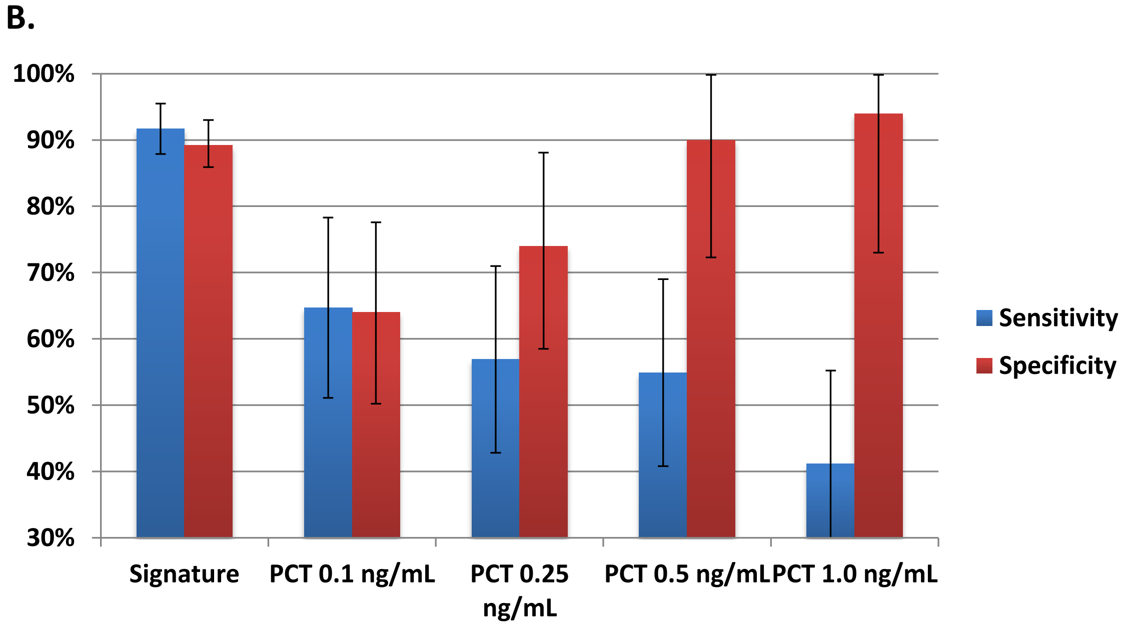

Another protein biomarker used in the clinical setting is the C-reactive protein (CRP), an acute phase response protein that is up-regulated in infections and other inflammatory conditions [1]. We compared the performance of CRP to that of the signature using the

entire study cohort (bacterial, viral) and Unanimous sub-cohort (bacterial, viral). CRP accuracy was determined using several standard cutoffs applied in the clinical setting [7–9]. Maximal CRP sensitivity of 92% was attained at 20 mg/mL cutoff resulting in a specificity of 60% (for the Unanimous sub-cohort [bacterial, viral]) (Fig. S13A). The signature had a similar sensitivity (94%) and a significantly higher specificity (93%, $P < 10^{-9}$) in the same cohort. Similar results were observed using the entire study cohort (bacterial, viral) (Fig. S13B). Overall, the signature has a similar sensitivity to CRP with a 20 mg/L cutoff but a considerably higher specificity for distinguishing bacterial from viral patients.

Figure S13. Comparison of the performance of the signature and CRP using different cutoffs. A. Performance measured in the Unanimous sub-cohort (bacterial, viral; n=527); B. Performance measured in the entire study cohort (bacterial, viral; n=653). Error bars represent 95% CI. Signature sensitivity and specificity were calculated after filtering out 14% of the patients with a marginal immune response.

A.

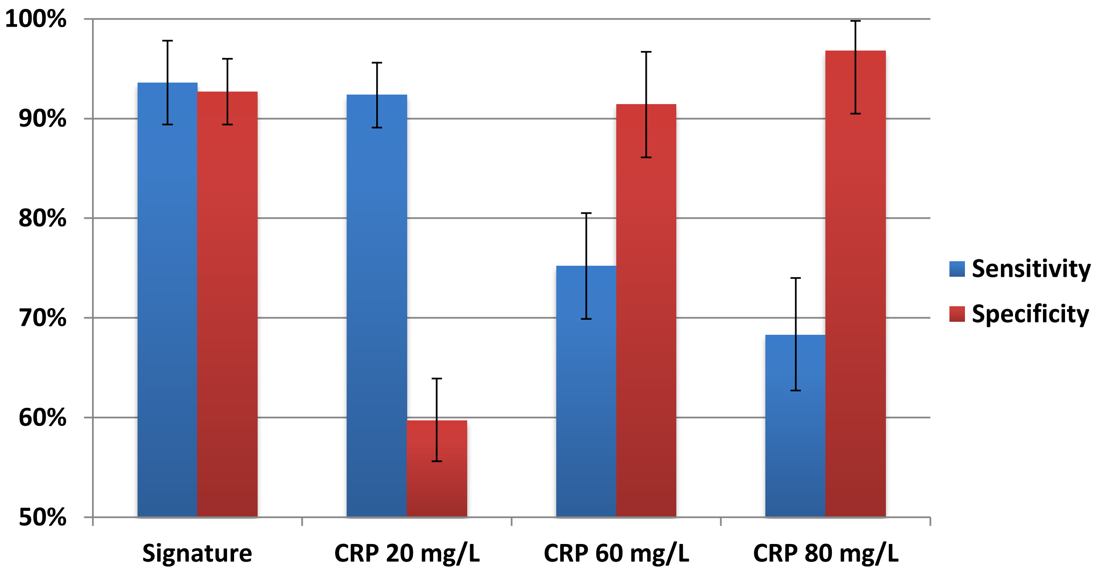

B.

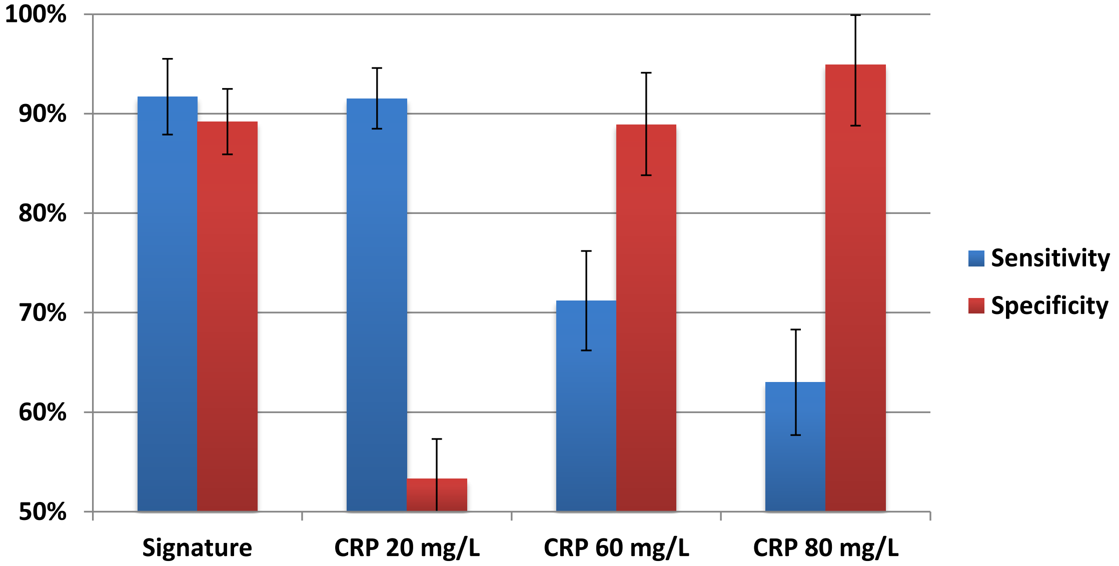

Next, we examined the differential response of protein biomarkers with a well-established role in the host response to infections (Table S10 and Fig. S14). Each biomarker was tested on at least 43 patients (about half bacterial and half viral), and if it showed promising results, it was further tested on additional patients (up to 150).

Table S10. A list of protein biomarkers with a well-established role in the host response against infections, and the number of patients used to test each biomarker (for each analysis the analyzed patients included approximately half bacterial and half viral patients).

Protein biomarker	Short description	No. of patients
CD11a	CD11a is expressed by all leukocytes as part of the integrin lymphocyte function-associated antigen-1 (LFA-1). LFA-1 plays a central role in leukocyte intercellular adhesion through interactions with its ligands, ICAMs 1-3 (intercellular adhesion molecules 1 through 3). CD11a also functions in lymphocyte co-stimulatory signaling.	120
CD11C	CD11C is an integrin α X chain protein and mediates cell-cell interactions during inflammatory responses.	79
CD80	CD80 is a membrane receptor involved in the co-stimulatory signal essential for T-lymphocyte activation. The binding of CD28 or CTLA-4 to CD80 induces T-cell proliferation and cytokine production.	82
HLA-A,B,C	These are MHC class I antigens associated with β2-microglobulin and are expressed by all human nucleated cells. HLA-A,B,C are central in cell-mediated immune response and tumor surveillance.	65
IFN-γ	IFN-γ is a soluble cytokine. IFN-γ participates in innate and adaptive immunity against viral and intracellular bacterial infections and in tumor control.	49
IL-1a	IL-1a is a member of the IL-1 cytokine family. IL-1a is a pleiotropic cytokine involved in various immune responses, inflammatory processes, and hematopoiesis. IL-1a is produced by monocytes and macrophages as a proprotein, which is proteolytically processed and released in response to cell injury, thereby inducing apoptosis.	43
IL-2	IL-2 is produced by T-cells in response to antigenic or mitogenic stimulation. IL-2 is required for T-cell proliferation and other activities crucial for regulation of the immune response.	49
IL-6	IL-6 is a cytokine that functions in inflammation and maturation of B cells. IL-6 is an endogenous pyrogen capable of inducing fever in people with autoimmune diseases or infections.	43
IL-8	IL-8 is a member of the CXC chemokine family and functions as one of the major mediators of the inflammatory response.	43
IL-9	IL-9 is a cytokine that acts as a regulator of a variety of hematopoietic cells. IL-9 supports IL-2 independent and IL-4 independent growth of helper T-cells.	43
IL-10	IL-10 is a cytokine produced primarily by monocytes and to a lesser extent by lymphocytes. IL-10 has pleiotropic effects in immunoregulation and inflammation.	48
IL-15	IL-15 is a cytokine that stimulates the proliferation of T-lymphocytes.	49
IL-16	IL-16 functions as a chemo-attractant, a modulator of T cell activation, and an inhibitor of HIV replication.	49
sTNFRSF1A	sTNFRSF1A is a receptor for TNFSF2/TNF-α and homo-trimeric TNFSF1/lymphotoxin-α that contributes to the induction of non-cytocidal TNF effects including anti-viral state and activation of the acid	54

	sphingomyelinase.	
TNF-α	TNF-α is a cytokine secreted mainly by macrophages. TNF-α can induce cell death of certain tumor cell lines. It is a potent pyrogen causing fever directly or by stimulation of IL-1 secretion.	43
TNF-β	TNF-β is a potent mediator of inflammatory and immune responses. It is produced by activated T and B lymphocytes and is involved in the regulation of various biological processes including cell proliferation, differentiation, apoptosis, lipid metabolism, coagulation, and neurotransmission.	43
TREM	TREM is a pro-inflammatory amplifier present on neutrophils and monocytes.	150

Figure S14. Box plots of levels of selected protein biomarkers (arbitrary units) in bacterial and viral patients. Red line and circle correspond to group median and average respectively. T-test p-values between bacterial and viral groups are depicted.

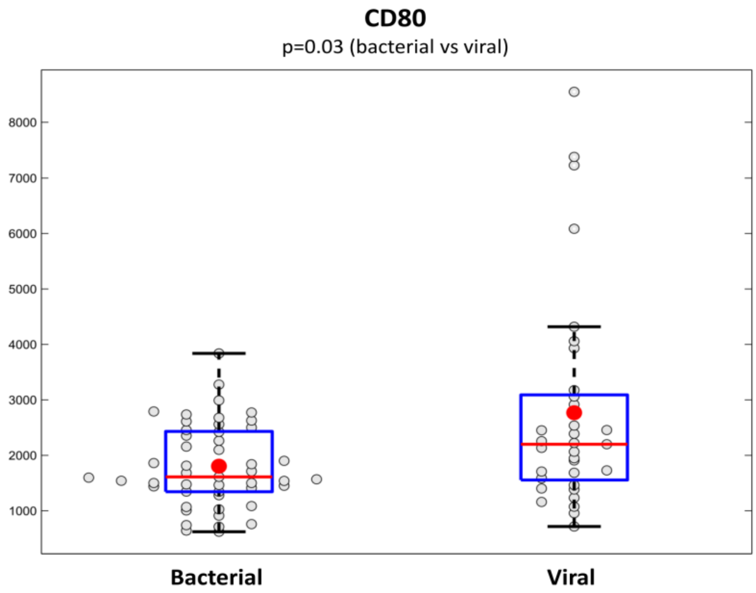

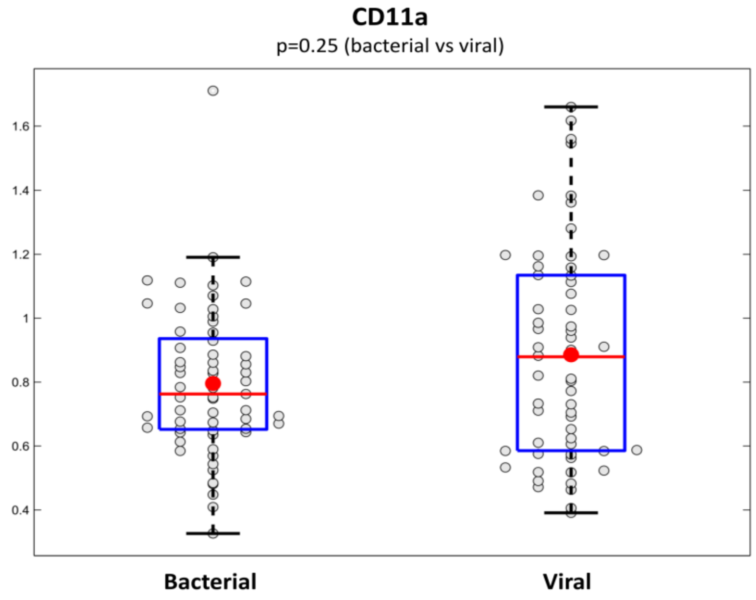
CD11a
p=0.25 (bacterial vs viral)
1.6
1.4
1.2
1
0.8
0.6
0.4
Bacterial
Viral

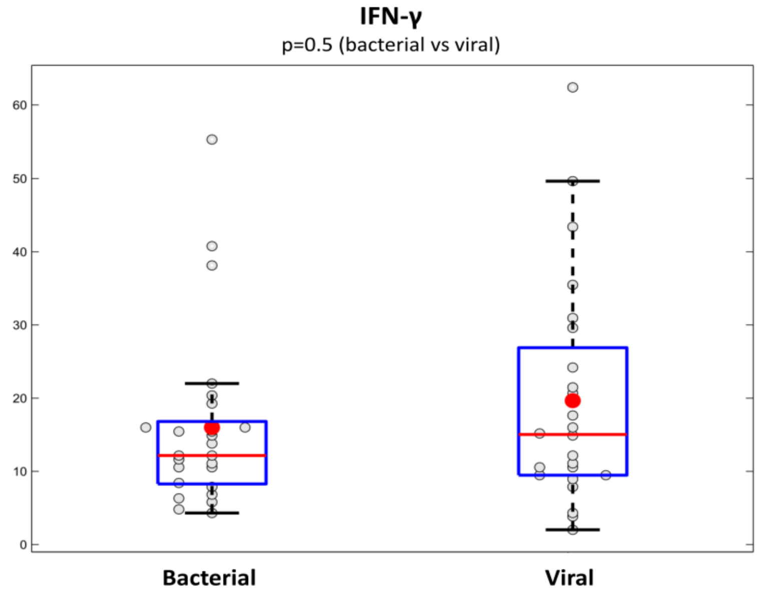
IFN-γ
p=0.5 (bacterial vs viral)
60
50
40
30
20
10
0
Bacterial
Viral

IL-2

p=0.42 (bacterial vs viral)

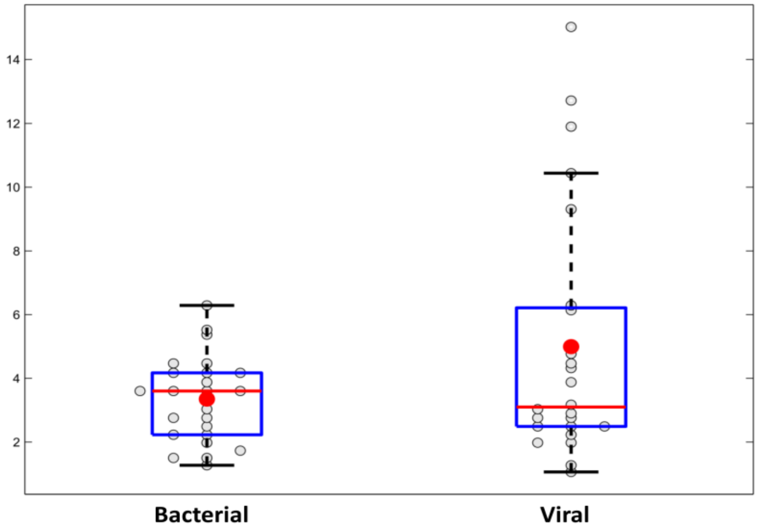

IL-10

p=0.04 (bacterial vs viral)

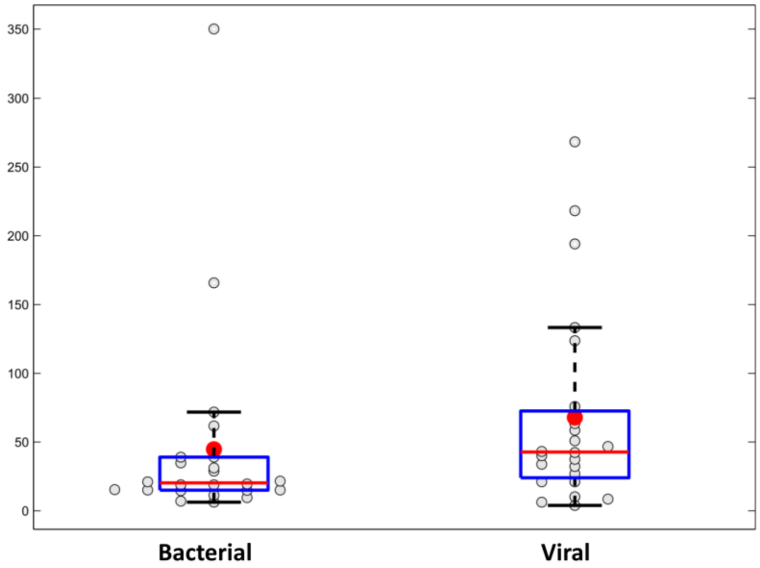

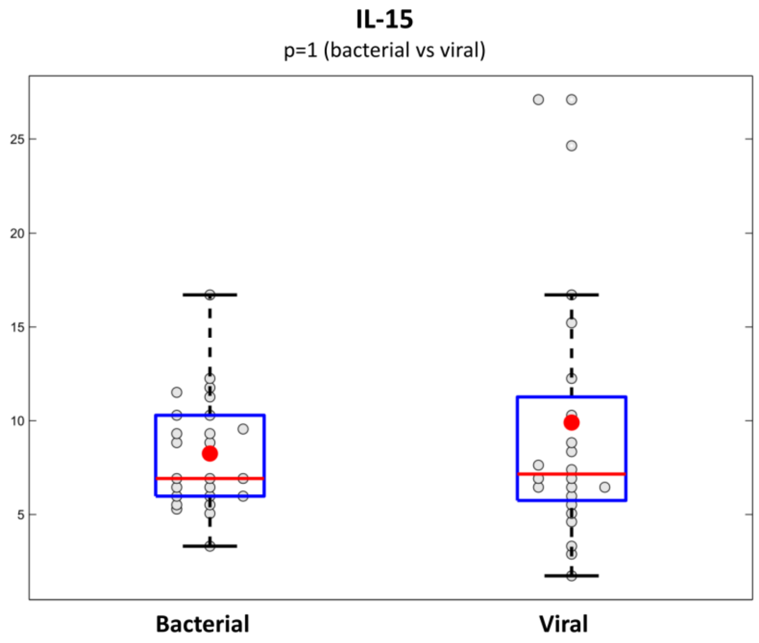
IL-15
p=1 (bacterial vs viral)
25
20
15
10
5
Bacterial
Viral

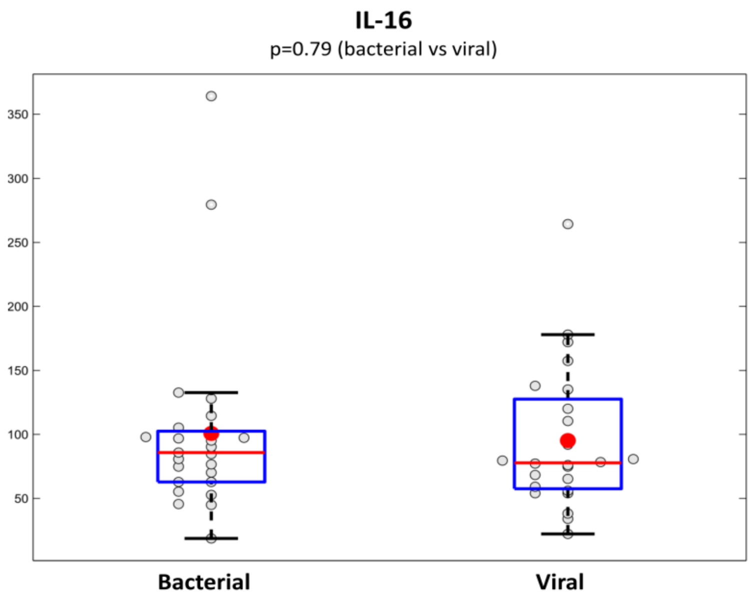
IL-16
p=0.79 (bacterial vs viral)
350
300
250
200
150
100
50
Bacterial
Viral

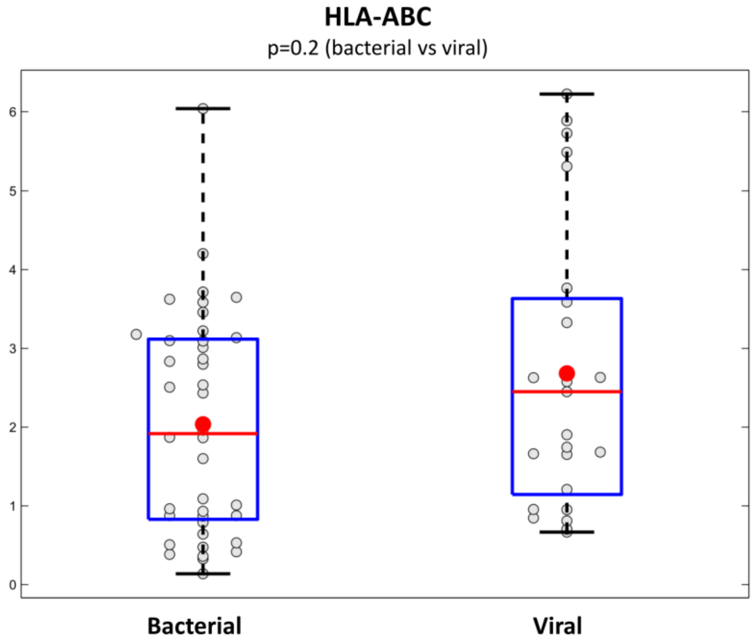

Since these biomarkers do not have a well-established cutoff in the clinical setting, we used their AUCs as a basis for comparison (Fig. 3B). The most informative biomarker was TREM (AUC of 0.68 ± 0.09). The accuracy of TREM was significantly lower than that of the signature ($P < 10^{-9}$ when comparing the two AUCs; Fig. 3B). These results demonstrate that mere participation of a protein in the host response to an infection does not necessarily imply diagnostic utility. For example, although IFN-γ has a well-established role in the immune response to viruses and intra-cellular bacteria, its short half-life (<20 h) [10] limits its diagnostic utility (as its concentration in the blood is highly dependent on the time from infection onset).

References

1. Reinhart K, Meisner M (2011) Biomarkers in the Critically Ill Patient: Procalcitonin. Critical Care Clinics 27: 253–263. doi:10.1016/j.ccc.2011.01.002.

2. Galetto-Lacour A, Zamora SA, Gervaix A (2003) Bedside procalcitonin and C-reactive protein tests in children with fever without localizing signs of infection seen in a referral center. Pediatrics 112: 1054–1060.

3. Christ-Crain M, Jaccard-Stolz D, Bingisser R, Gencay MM, Huber PR, et al. (2004) Effect of procalcitonin-guided treatment on antibiotic use and outcome in lower respiratory tract infections: cluster-randomised, single-blinded intervention trial. Lancet 363: 600–607. doi:10.1016/S0140-6736(04)15591-8.

4. Hsiao AL, Baker MD (2005) Fever in the new millennium: a review of recent studies of markers of serious bacterial infection in febrile children. Curr Opin Pediatr 17: 56–61.

5. Christ-Crain M, Stolz D, Bingisser R, Müller C, Miedinger D, et al. (2006) Procalcitonin guidance of antibiotic therapy in community-acquired pneumonia: a randomized trial. Am J Respir Crit Care Med 174: 84–93. doi:10.1164/rccm.200512-1922OC.

6. Dubos F, Korczowski B, Aygun DA, Martinot A, Prat C, et al. (2008) Serum procalcitonin level and other biological markers to distinguish between bacterial and aseptic meningitis in children: a European multicenter case cohort study. Arch Pediatr Adolesc Med 162: 1157–1163. doi:10.1001/archpedi.162.12.1157.

7. Enguix A, Rey C, Concha A, Medina A, Coto D, et al. (2001) Comparison of procalcitonin with C-reactive protein and serum amyloid for the early diagnosis of bacterial sepsis in critically ill neonates and children. Intensive Care Medicine 27: 211–215. doi:10.1007/s001340000709.

8. Fernández Lopez A, Luaces Cubells C, García García JJ, Fernández Pou J (2003) Procalcitonin in pediatric emergency departments for the early diagnosis of invasive bacterial infections in febrile infants: results of a multicenter study and utility of a rapid qualitative test for this marker. Pediatr Infect Dis J 22: 895–903. doi:10.1097/01.inf.0000091360.11784.21.

9. Flanders SA, Stein J, Shochat G, Sellers K, Holland M, et al. (2004) Performance of a bedside C-reactive protein test in the diagnosis of community-acquired pneumonia in adults with acute cough. Am J Med 116: 529–535. doi:10.1016/j.amjmed.2003.11.023.

10. Schoenborn JR, Wilson CB (2007) Regulation of interferon-gamma during innate and adaptive immune responses. Adv Immunol 96: 41–101. doi:10.1016/S0065-2776(07)96002-2.
